# Knock-in mice expressing a humanized arachidonic acid 15-lipoxygenase (Alox15) carry a partly dysfunctional erythropoietic system

**DOI:** 10.1186/s11658-023-00511-3

**Published:** 2023-11-29

**Authors:** Florian Reisch, Dagmar Heydeck, Marjann Schäfer, Michael Rothe, Jiaxing Yang, Sabine Stehling, Gerhard P. Püschel, Hartmut Kuhn

**Affiliations:** 1 Department of Biochemistry, Charité–Universitätsmedizin Berlin, corporate member of Freie Universität Berlin and Humboldt Universität Zu Berlin, Charitéplatz 1, 10117 Berlin, Germany; 2 Institute for Nutritional Sciences, University of Potsdam, Arthur-Scheunert-Allee 114-116, 14558 Nuthetal, Germany; 3 Lipidomix GmbH, Robert-Rössle-Straße 10, 13125 Berlin, Germany

**Keywords:** Eicosanoids, Lipid peroxidation, Oxidative stress, Polyenoic fatty acids, Erythropoiesis

## Abstract

Arachidonic acid 15-lipoxygenases (ALOX15) play a role in mammalian erythropoiesis but they have also been implicated in inflammatory processes. Seven intact *Alox* genes have been detected in the mouse reference genome and the mouse *Alox15* gene is structurally similar to the orthologous genes of other mammals. However, mouse and human ALOX15 orthologs have different functional characteristics. Human ALOX15 converts C_20_ polyenoic fatty acids like arachidonic acid mainly to the n-6 hydroperoxide. In contrast, the n-9 hydroperoxide is the major oxygenation product formed by mouse Alox15. Previous experiments indicated that Leu353Phe exchange in recombinant mouse Alox15 humanized the catalytic properties of the enzyme. To investigate whether this functional humanization might also work in vivo and to characterize the functional consequences of mouse Alox15 humanization we generated *Alox15* knock-in mice (*Alox15-KI*), in which the Alox15 gene was modified in such a way that the animals express the arachidonic acid 15-lipoxygenating Leu353Phe mutant instead of the arachidonic acid 12-lipoxygenating wildtype enzyme. These mice develop normally, they are fully fertile but display modified plasma oxylipidomes. In young individuals, the basic hematological parameters were not different when *Alox15-KI* mice and outbred wildtype controls were compared. However, when growing older male *Alox15-KI* mice develop signs of dysfunctional erythropoiesis such as reduced hematocrit, lower erythrocyte counts and attenuated hemoglobin concentration. These differences were paralleled by an improved ex vivo osmotic resistance of the peripheral red blood cells. Interestingly, such differences were not observed in female individuals suggesting gender specific effects. In summary, these data indicated that functional humanization of mouse Alox15 induces defective erythropoiesis in aged male individuals.

**Graphical Abstract:**

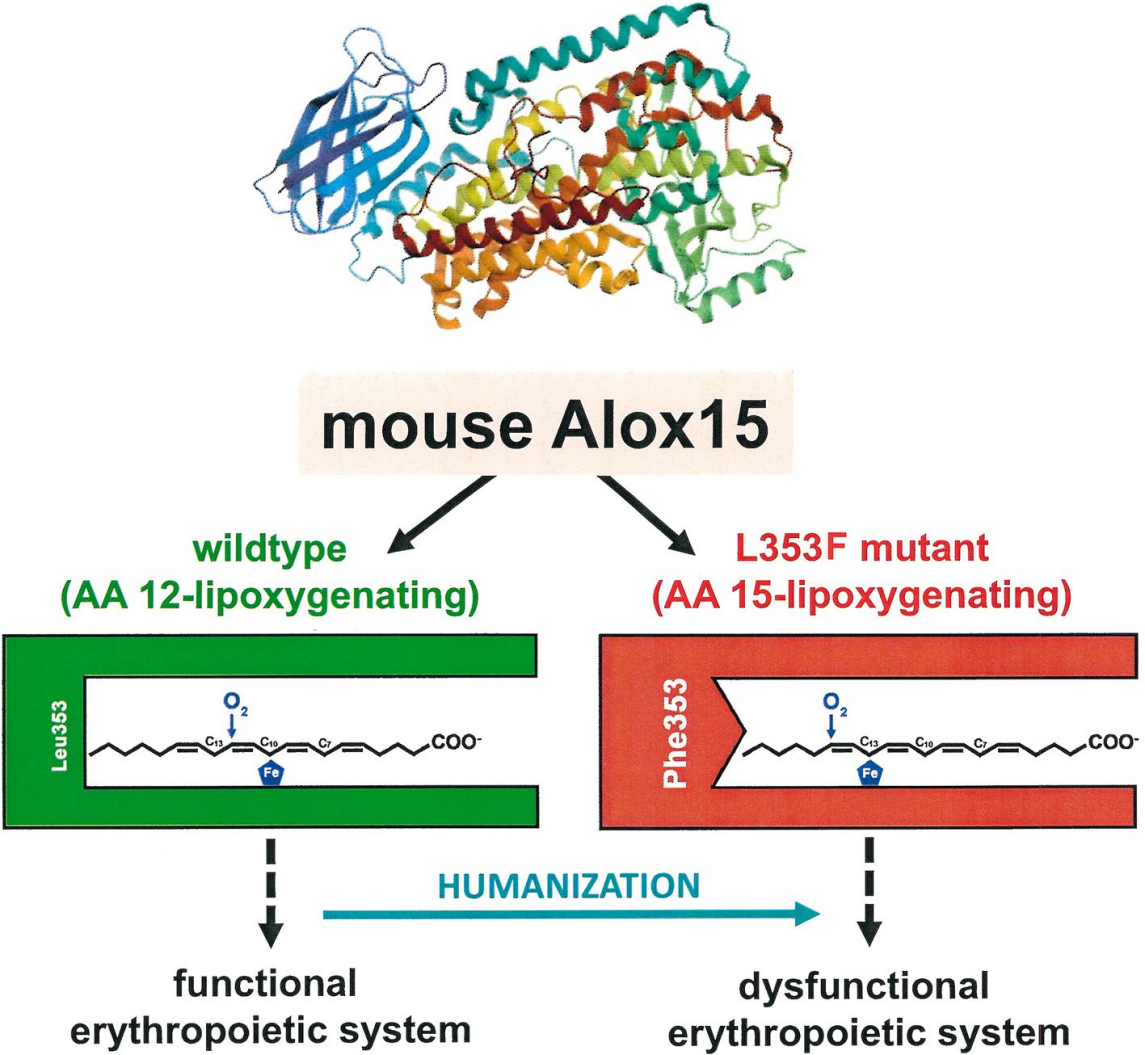

**Supplementary Information:**

The online version contains supplementary material available at 10.1186/s11658-023-00511-3.

## Background

In our aerobic world lipid peroxidation occurs in most living systems and many physiological and patho-physiological processes are affected [1, 2]. Arachidonic acid (AA) lipoxygenases (ALOX-isoforms) are lipid peroxidizing enzymes that are widely distributed in highly developed animals [3] and plants [4]. They play a role in cell differentiation and in the pathogenesis of human diseases [5–8]. In humans six functional *ALOX* genes are present and except for the *ALOX5* gene all other *ALOX* genes are found on chromosome 17 [9]. In mice, single copy orthologous genes exists for each human ALOX isoform but in addition, there is a functional *Aloxe12* gene that is a corrupted pseudogene in the human genome [9]. Although the amino acid sequences of mouse and human ALOX15 orthologs are very similar (> 80% sequence identity) the enzymes have different functional properties. The major AA oxygenation product of mouse Alox15 is 12S-H(p)ETE [10, 11] whereas human ALOX15 produces mainly 15S-H(p)ETE [12]. The structural reasons for the distinct catalytic characteristics of the two enzymes have been investigated in the past and the Triad Concept [13, 14] was developed. According to this hypothesis three clusters of amino acids (Phe353, Ile418 + Met419, Ile593) limit the bottom of the substrate binding pocket of mammalian ALOX15 orthologs and the geometry of these residues determines the position of the fatty acid substrate at the catalytic center [14, 15]. When space-filling amino acids are located at these positions as it is the case for AA 15-lipoxygenating ALOX15 orthologs, fatty acid substrates are prevented from penetrating deeply into the substrate binding crevice so that AA 12-lipoxygenation is prevented. Instead, hydrogen is abstracted from C_13_ and thus, AA 15-lipoxygenation is catalyzed [14, 15]. If small amino acids occupy the triad positions, AA slides deeper into the substrate binding cleft and thus, AA C_12_ oxygenation is catalyzed [14, 15]. When Phe353 of human ALOX15 is mutated in vitro to Leu (Phe353Leu), the AA 15-lipoxygenating enzyme is converted to an AA 12-lipoxygenating protein [16]. An inverse mutagenesis strategy using the AA 12-lipoxygenating mouse Alox15 (Leu353Phe) humanized the reaction specificity of this enzyme [16]. All mammalian ALOX15 orthologs tested so far [17, 18] follow the Triad Concept.

The biological roles of mammalian ALOX15 orthologs are still a matter of discussion [5, 7]. Rabbit ALOX15 is high level expressed in late reticulocytes and oxidizes mitochondrial membrane lipids and thus, contributes to the maturational decline of cellular respiration during red blood cell maturation [19–21]. However, *Alox15*^*−/−*^ mice are viable and do not display major signs of dysfunction of the erythropoietic system [11]. More recently, we re-explored the impact of functional inactivation of the *Alox15* gene on red blood cell development and observed that the erythropoietic system of *Alox15*^*−/−*^ mice is indeed compromised [22]. Although the major red blood cell parameters of *Alox15*^*−/−*^ mice were in the normal range, we observed significantly lower red blood cell counts, impaired hematocrit values and reduced hemoglobin concentrations. Introduction of a human *ALOX15* transgene, the expression of which is controlled by the aP2 promoter, normalized the impaired erythropoietic parameters and thus, rescued the defective erythropoietic phenotype [22].

As indicated above Leu353Phe exchange altered the reaction specificity of recombinant mouse Alox15 and forced in vitro humanization of this enzyme property [16]. To investigate whether this mutagenesis strategy does also work in vivo and to analyze the functional consequences of this subtle genetic manipulation under in vivo conditions we generated knock-in mice (*Alox15*-*KI* mice) expressing the Leu353Phe mutant instead of wildtype ALOX15. We found that *Alox15*-KI mice are viable, fully fertile and developed normally up to an age of 75 weeks. They showed interesting differences in their plasma oxylipidomes, which mirror the genetic manipulation on the level of the plasma lipids. When aging, male but not female *Alox15*-KI individuals displayed impaired erythrocyte parameters and the osmotic resistance of their red blood cells was modified. Based on this data we concluded that functional humanization of mouse Alox15 compromised erythropoiesis in a gender-specific manner.

## Methods

### Chemicals

Chemicals used for this study were of following origin: Arachidonic acid (AA) and standards of 15-HETE, 12-HETE, 5-HETE, 12-HEPE, 15-HEPE, 14-HDHA, 17-HDHA, 13-HODE, 13-HOTrE) from Cayman Chem.; sodium borohydride from Life Technologies, Inc. (Eggenstein, Germany); restriction enzymes from ThermoFisher (Schwerte, Germany); isopropyl-β-thiogalactopyranoside (IPTG) from Carl Roth GmbH (Karlsruhe, Germany); *E. coli* (strain Rosetta2 DE3 pLysS) from Novagen (Merck-Millipore, Darmstadt, Germany). Oligonucleotide synthesis was carried out at BioTez Berlin Buch GmbH (Berlin, Germany). Nucleic acid sequencing was performed at Eurofins MWG Operon (Ebersberg, Germany). HPLC grade solvents and water were purchased from Fisher Scientific (New Hampshire, USA). The origin of other chemicals employed in this study is specified in the description of the methods. The following chemicals were used for oxylipidomic measurements: Deuterated standards (LTB4-d4, 20-HETE-d6, 15-HETE-d8, 13-HODE-d4, 14,15-DHET-d11, 9,10-DiHOME-d4, 12,13-EpOME-d4, 8,9-EET-d11, PGE2-d4; 10 ng/ml each) from Cayman Chem. (Ann Arbor, USA); acetonitrile, solvents from Merck (Darmstadt, Germany) and Fisher Scientific (Schwerte, Germany).

### Bacterial expression of Alox15 variants

Recombinant Alox15 orthologs were expressed in *E. coli* as described in [18] and methodological details of the expression procedure are given in the Additional file 1.

### Site directed mutagenesis

The PfuUltra II Hotstart PCR Master Mix kit (Agilent Technologies, Waldbronn, Germany) was employed to humanize the functional characteristics of mouse Alox15. For this purpose we followed the experimental protocol described in [18] and the methodological details of the mutagenesis procedure including the sequence of the mutagenesis primers are given in the Additional file 1.

### In vitro* ALOX15 *activity assays

The activity of recombinant enzymes was determined adding different amounts of the bacterial lysate supernatants containing the recombinantly expressed enzyme to a reaction volume of 0.5 ml PBS supplemented with 100 µM arachidonic acid. After an 8 min incubation period the amounts of oxygenation products were quantified by RP-HPLC. The methodological details of the in vitro activity assays are given in the Additional file 1.

### Generation of Alox15-KI mice and off-target analysis

The Alox15-KI mice characterized here were created in collaboration with Cyagen Bioscience (Santa Clara, USA). For this purpose, we employed a similar strategy that was used to humanize the reaction specificity of mouse Alox15b [23] but the protocol was adopted to mouse Alox15. The experimental details are explained in the Methodological Additional file 1. The animal experiments carried out in this study were approved by the local Animal Care Committee (Landesamt für Gesundheit und Soziales, Berlin, Germany, T0437/08).

### Analyses of potential off-target alteration

In general, gRNA mediated Crispr-Cas9 in vivo mutagenesis is very specific but occasionally unintended off-target site-directed mutagenesis might occur. To test whether our strategy has introduced off-target alterations we first screened in silico the mouse reference genome for the targeting sequence GAA GCT GTA AGT CTG AGC TTC GG. Here we identified five possible off-target sites. These genomic regions were amplified for several individuals of our *Alox15*-KI mouse colony by genomic PCR and the nucleotide sequence of the amplification products did only reveal wildtype sequences (Additional file 1: Table S1).

### Genotyping

To explore whether the wanted mutation was introduced during Crispr/Cas9 in vivo mutagenesis PCR genotyping was carried out and the following primers were employed. (i) forward primer: TAG GCA CCC AGG TAG GCT TTG GT, (ii) reverse primer: CTG AAC CCT GCC TGA GAT GCC AC. For both, the wildtype and the mutant alleles a 580 bp PCR fragment was sequenced.

### Quantitative RT-PCR of different Alox-isoforms in peritoneal lavage cells.

Peritoneal lavage cells were prepared as described below and total RNA extraction as well as cDNA synthesis was performed as described in [24]. Experimental details of qRT-PCR including the primer combinations used for the different mouse Alox-isoforms and the amplification protocol are described in the Methodological supplement (Additional file 1: Table S2).

### Breeding of Alox15-KI mice and outbred wildtype controls

Heterozygous *Alox15*-KI mice were obtained as described above. These heterozygous individuals were inter-crossed and homozygous *Alox15*-KI individuals as well as outbred wildtype control animals were selected and separate colonies of wildtype controls and homozygous *Alox15*-KI mice were established for comparative experiments. Inter-colony cross-breeding was avoided and each individual animal of the two colonies was genotyped.

### Preparation of peritoneal lavage cells

Mice of either genotype *(Alox15*-KI mice, outbred wildtype controls) were sacrificed under isoflurane anesthesia. The peritoneal cavity was rinsed with 10 ml of pre-warmed PBS. The rinsing fluid was aspirated, the cells were pelleted by centrifugation (500×*g* for 15 min), washed and resuspended in PBS to reach a density of about 5 × 10^6^ cells per ml. After cell counting 0.5 ml aliquots of this suspension were used for ex vivo ALOX15 activity assays or for total RNA extraction.

### Preparation of bone marrow cells

Mice of either genotype (*Alox15-KI* mice, outbred wildtype control mice) were sacrificed, the femur bones were isolated and the bone marrow cavity was rinsed with 10 ml of PBS. The cell suspension was filtered to remove bone fragments, the cells were pelleted at 500×*g*, washed and redissolved in PBS reaching a density of about 5 × 10^6^ cells per ml. Aliquots of this suspension were employed for ex vivo ALOX15 activity assays.

### Ex vivo activity assays using peritoneal lavage and bone marrow cells

For ex vivo activity assays 0.5 ml of a cell suspension of peritoneal lavage cell or bone marrow cell suspension (2–10 × 10^6^ cells per ml PBS) were incubated for 5–10 min in PBS containing 100 µM AA. After the incubation period the AA oxygenation products were quantified using RP-HPLC. The methodological details of sample workup and of the analytical strategy are described in detail the Additional file 1: Methodological supplement.

### Quantification of hematological parameters

Basic hematological parameters [whole blood hemoglobin content (Hb), hematocrit values (Hk), erythrocyte count (erys), leukocyte count (leukos), mean corpuscular volume (MCV), mean corpuscular hemoglobin content (MCH), mean corpuscular hemoglobin concentration (MCHC)] were quantified for six randomly selected individuals (either sex) of the two genotypes in two different age categories (young mice, 10–20 weeks; old mice, 70–75 weeks) at the Institut für Veterinärmedizinische Diagnostik GmbH (Berlin, Germany).

### Quantification of bodyweights

Body weight kinetics of *Alox15*-KI mice (either sex) and of wildtype controls (*n* = 10) were quantified as described previously [23]. Statistic evaluation of the experimental raw data was performed using two-way ANOVA.

### Erythrocyte osmotic resistance

Dysfunctional erythrocytes rapidly undergo osmotic hemolysis [25] and we followed the experimental protocol described in [22] to compare the osmotic resistance of the red blood cells of *Alox15*-KI mice and of outbred wildtype control animals. The methodological details of blood collection, sample workup and hemolysis measurements are described in detail in the Additional file 1: Methodological supplement.

### Blood plasma oxylipidomics

To investigate, whether the altered reaction specificity of mouse Alox15 might have altered the pattern of plasma oxylipins we quantified more than 40 different free oxygenated PUFAs in the blood plasma [26]. The methodological details of sample workup and of the analysis procedure [23, 26] are described in the Additional file 1: Methodological supplement including Table S3, Table S4, Table S5 and Table S6.

### Ex vivo* Alox5 *activity assay

200 µl of heparinized blood was prepared from *Alox15*-KI mice and corresponding wildtype control animals (*n* = 5 for each genotype). 5 µM of calcium ionophore A23187 (in DMSO) was added and the suspension was incubated for 15 min at 37 °C. Subsequently, the formation of leukotriene B4 was measured by LC–MS/MS. The methodological details of sample workup and of the analysis procedure are described in the Additional file 1: Methodological supplement.

### Quantitative RT-PCR of Alox15 in different mouse tissues

RNA extraction, synthesis of cDNA and qRT-PCR were performed as described in [24] and the following amplification primers were used for mouse *Alox15* cDNA: forward, 5′-GTA CGC GGG CTC CAA CAA CGA-3′ and reverse, 3′-TCT CCG GGG CCC TTC ACA GAA-5′.

### Statistics

Experimental raw data were statistically evaluated with the two-sided Student’s *t*-test using the Microsoft Excel software package (Excel 2016) or with the Mann–Whitney U-test using the GraphPad prism program. For statistical evaluation of the body weight kinetics and of the osmotic hemolysis curves we used the two-way ANOVA function of the GraphPad Prism software package, version 8.2.0 (GraphPad Software, San Diego, USA). This program was also employed to visualize the experimental data.

## Results

### Leu353Phe exchange of mouse Alox15 humanized the specificity of AA oxygenation

**Fig. 1:**
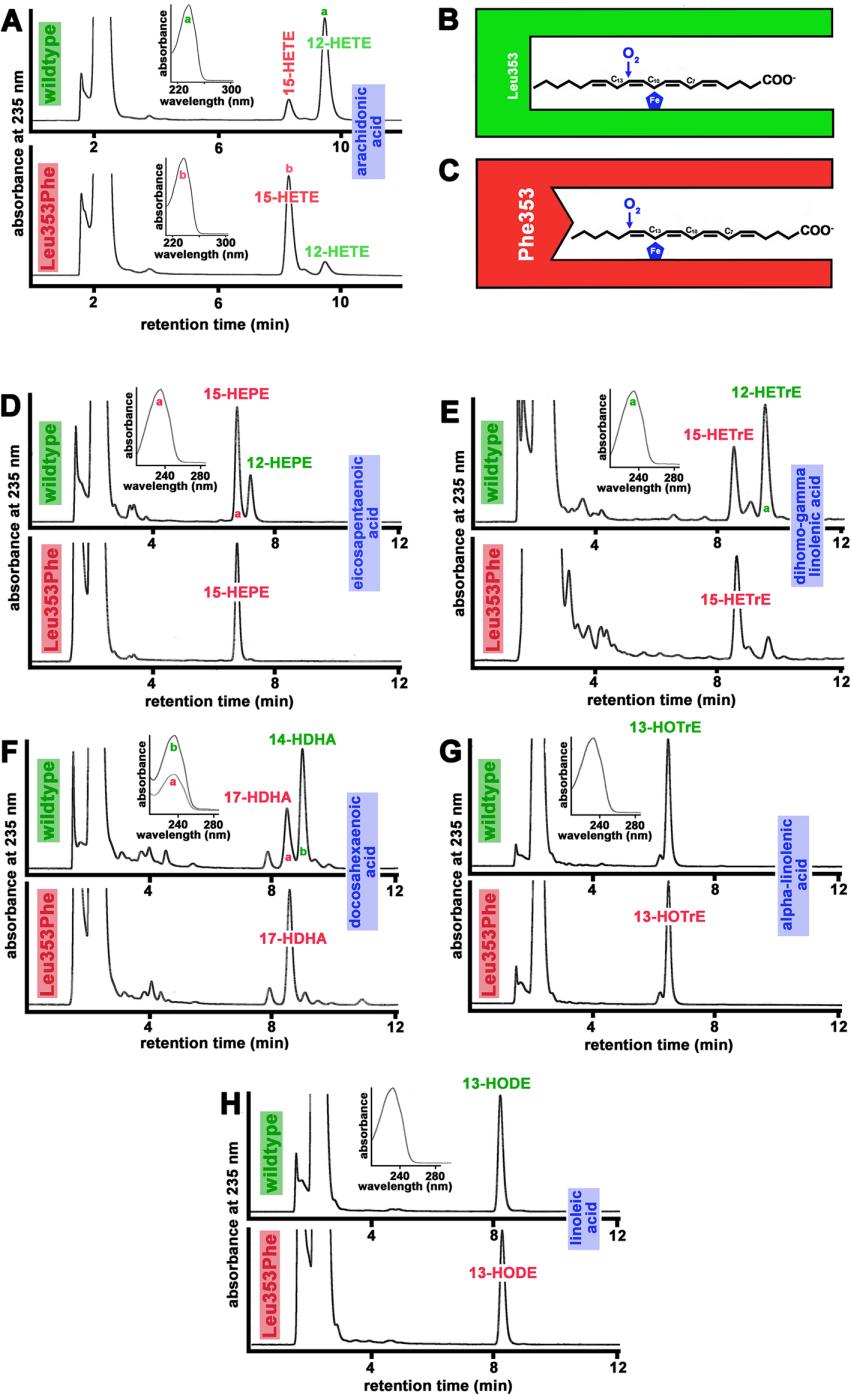
Reaction specificity of wildtype and mutant recombinant mouse Alox15 with different polyenoic fatty acids. Wildtype and mutant (Leu353Phe) mouse Alox15 were expressed as N-terminal his-tag fusion proteins as described in the Additional file 1 and aliquots of the bacterial lysis supernatants were used as enzyme source. After a 5 min incubation period with different polyenoic fatty acids (100 µM) the reaction products were analyzed by RP-HPLC. **A** AA oxygenation products. **B** Triad Concept cartoon explaining AA 12-lipoxygenation by wildtype mouse Alox15 (see text for detailed explanation). **C** Triad Concept cartoon explaining AA 15-lipoxygenation by the Leu353Phe mutant of mouse Alox15 (see text for detailed explanation). **D** EPA oxygenation products, **E** dihomo-gamma-linoleic acid oxygenation products, **F** DHA oxygenation products, **G** alpha-linolenic acid oxygenation products, **H** linoleic acid oxygenation products. Insets: UV-spectra of the major conjugated dienes peak labeled by small letters. Representative chromatograms (*n* ≥ 4) are shown

To prove that Leu353Phe exchange humanized the reaction specificity of mouse Alox15 [16] wildtype mouse Alox15 and its Leu353Phe mutant were recombinantly expressed in *E. coli* and in vitro activity assays were carried out. As indicated in Fig. 1A (upper panel) 12-HETE was identified as major conjugated diene. The chemical identity of the two major HPLC peaks was identified by co-chromatography of authentic standards and uv-spectroscopy indicating a classical conjugated diene spectrum for the major oxygenation product (inset to Fig. 1A, upper panel).

In Fig. 1B a schematic presentation of substrate binding at the active site of wildtype mouse Alox15 is given. Substrate fatty acids slide into the catalytic center of the enzyme with its methyl end ahead and the deepness of the catalytic center is limited by the side chains of the triad amino acids [13, 14]. For mouse Alox15 Leu353 is the functionally most important triad residue [27]. Thus, arachidonic acid is aligned at the catalytic center of the enzyme in such a way that the bisallylic methylene C_10_ is located close to the non-heme iron, which allows hydrogen abstraction from C_10_. Dioxygen is subsequently introduced at C_12_ ([+ 2] radical rearrangement), explaining the major formation of 12-HETE. The major reaction product of the Leu353Phe mutant was 15-HETE (Fig. 1A, lower panel) and here again the uv-spectrum (inset to Fig. 1A, lower panel) was consistent with the chemical structure of the two major AA oxygenation products (12-HETE, 15-HETE). In other words, Leu353Phe mutation humanized the reaction specificity of recombinant mouse Alox15 for AA oxygenation. In Fig. 1C a schematic presentation of substrate binding at the active site of the mutant enzyme is shown. Since Phe carries a more bulky and less flexible side chain than Leu353 the substrate binding pocket has a reduced volume so that AA cannot penetrate as deeply into the cavity. In this enzyme–substrate complex hydrogen abstraction from the bisallylic methylene C_10_ is sterically hindered. Instead, the bisallylic methylene C_13_ is now located close to the non-heme iron and thus, hydrogen is removed from this carbon atom. Consequently, oxygen may be introduced at C_15_ ([+ 2] radical rearrangement), which explains the major formation of 15-HETE by the Leu353Phe Alox15 mutant. In summary, Leu353Phe exchange humanized the reaction specificity of recombinant mouse Alox15 for AA oxygenation and this functional alteration can be explained by the Triad Concept [13, 14].

### Reaction specificities of mouse Alox15 with other fatty acids as substrate are also humanized by Leu353Phe exchange

To interpret possible differences in the plasma oxylipidomes of *Alox15*-KI mice and wildtype controls, we next characterized the reaction specificities of recombinant wildtype Alox15 and its Leu353Phe mutant with other PUFAs. For the wildtype enzyme such data have previously been reported [28] but for the Leu353Phe mutant corresponding results have not been published.

5,8,11,14,17-eicosapentaenoic acid (EPA) is an AA derivative that carries an additional double bond at the omega-3 position. In theory, this omega-3 polyunsaturated fatty acid (PUFA) should be oxygenated by wildtype mouse Alox15 dominantly to 12-HEPE. When we tested this prediction experimentally, we found that wildtype mouse Alox15 oxygenated EPA mainly to 15-HEPE (70%) and 12-HEPE was only formed as minor (30%) side product (Fig. 1D, upper trace). Here again, the uv-spectrum of the major oxygenation product indicated a conjugated diene chromophore (inset to Fig. 1B, upper trace). These data are consistent with the outcome of previous experiments analyzing the reaction specificity of wildtype mouse Alox15 using EPA as oxygenation substrate [28]. For the Leu353Phe mutant (Fig. 1D, lower trace) almost exclusive formation of 15-HEPE was analyzed. The observed differences in the reaction specificity of EPA oxygenation between wildtype mouse Alox15 and its Leu353Phe mutant (elevated relative share of 15-HEPE) were predicted on the basis of the Triad Concept.

Dihomo-gamma-linolenic acid (DGL) is an AA derivative, which lacks the C_5_=C_6_ double bond. This fatty acid is oxygenated by wildtype mouse Alox15 to a 4:6 mixture of 15-HETrE and 12-HETrE (Fig. 1E, upper trace). When the Leu353Phe mutant was used for in vitro activity assays (Fig. 1E, lower trace), we also observed the formation of these oxygenation products but here the 15-HETrE / 12-HETrE ratio was about 8:2. The observed quantitative differences in the product pattern of wildtype and mutant mouse Alox15 (elevated relative share of 15-HETrE by the mutant enzyme) were also predicted on the basis of the Triad Concept.

4,7,10,13,16,19-Docosahexaenoic acid (DHA) is oxygenated by wildtype mouse Alox15 mainly to 14-HDHA [28] but under our experimental conditions we also observed significant amounts (30%) of 17-HDHA as DHA oxygenation product (Fig. 1F, upper trace). Both products are characterized by the canonical conjugated diene chromophore and their chemical identity was confirmed by co-chromatography with authentic standards. Leu353Phe exchange altered the reaction specificity in favor of 17-HDHA formation (Fig. 1F, lower trace) and this alteration was predicted on the basis of the Triad Concept.

Linoleic acid (LA) and alpha-linolenic acid (ALA) are C_18_-PUFAs, which lack n-11 bisallylic methylenes and thus, n-9 oxygenation (formation of 10-HODE from LA and 10-HOTrE from ALA) is not possible. These fatty acids are almost exclusively oxygenated by wildtype and mutant mouse Alox15 to the n-6 oxygenated derivatives (13-HOTrE, Fig. 1G, upper trace; 13-HODE, Fig. 1H, upper trace). For these PUFAs no alterations in the reaction specificity were induced by Leu353Phe exchange (Figs. 1G, H, lower traces).

### Peritoneal lavage cells and bone marrow cells are major sources of Alox15 expression

**Fig. 2:**
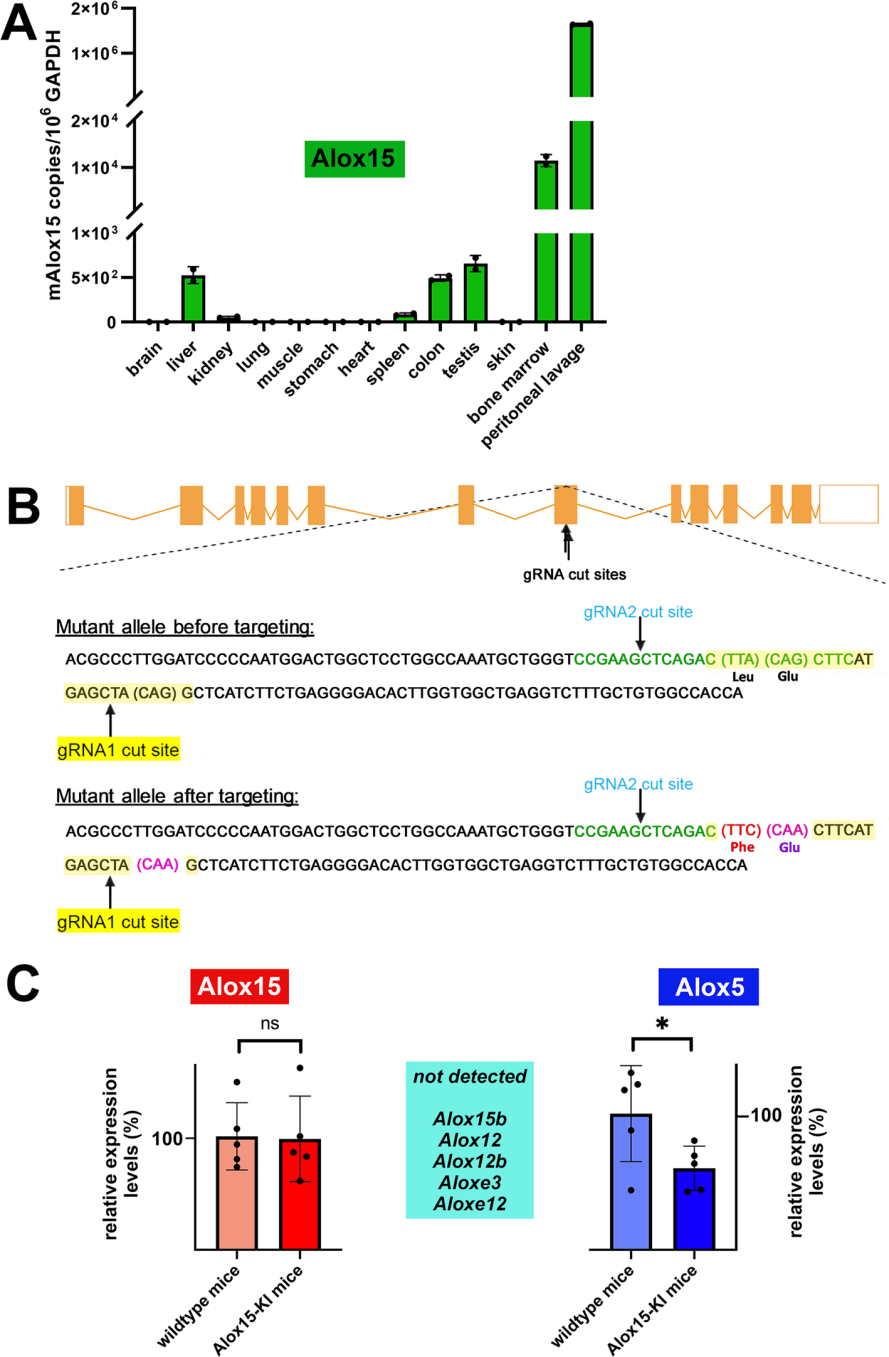
Expression of* Alox15* mRNA in different mouse cells and knock-in strategy for generation of humanized Alox15 knock-in mice. **A** Total RNA was extracted from different cells and tissues of wildtype mice, the RNA was reversely transcribed and the *Alox15* mRNA steady state concentrations were quantified by qRT-PCR (see Additional file 1: Methodological supplement). Two independent measurements (*n* = 2) were carried out for each cDNA sample. **B** Crisp/Cas9 strategy for generation *Alox15*-KI mice. **C** Quantitative RT-PCR of Alox-isoforms in peritoneal lavage cells prepared from Alox15-KI mice and outbred wildtype control animals. Expression levels of Alox15 and Alox5 mRNA in wildtype cells were separately set 100%. Experimental details are given in the Additional file 1: Methodological supplement

Human ALOX15 is constitutively expressed in large quantities in reticulocytes, airway epithelium and eosinophilic granulocytes [29]. Peripheral monocytes hardly express the enzyme but cell stimulation with interleukin-4 [30] or interleukin-13 [31] upregulated ALOX15 expression. In pigs, the enzyme is present in large amounts in peripheral leukocytes [32] but in mice the enzyme is most abundantly expressed in peritoneal lavage cells [10, 11]. To select suitable cell types for ex-vivo activity assays we first profiled *Alox15* mRNA expression in different mouse tissues by qRT-PCR. From Fig. 2A it can be seen, that hardly any *Alox15* mRNA was detected in RNA extracts of brain, lung, muscle, stomach, heart and skin. Low *Alox15* mRNA expression levels (< 300 *Alox15* mRNA copies per 10^6^ Gapdh mRNA copies) were detected in liver, kidney, spleen, colon and testis but high *Alox15* mRNA concentrations (> 10^4^ *Alox15* mRNA copies per 10^6^ Gapdh mRNA copies) were observed in bone marrow cells. The richest *Alox15* mRNA source were peritoneal lavage cells. In these cells, the *Alox15* mRNA expression levels did even exceed the Gapdh expression by a factor of 2. These data prompted us to employ peritoneal lavage cells as well as bone marrow cells for later ex-vivo Alox15 activity assays.

### Generation of Alox15 knock-in mice expressing the Leu353Phe Alox15 point mutant

To generate heterozygous knock-in mice that express the Alox15 Leu353Phe point mutant instead of the wildtype enzyme we employed the Crispr/Cas9 in vivo mutagenesis strategy as described in the Additional file 1: Methodological supplement. We first designed a gRNA targeting vector and a donor oligonucleotide that involved the target sequence flanked by homologous sequences at the 5′- and the 3′-ends. The Leu353Phe (TTA to TTC) mutation site in the donor oligonucleotide was introduced into exon 8 of the *Alox15* gene by homology-directed repair mechanisms. To prevent methodological artifacts an additional silent mutation (CAG to CAA) was introduced (Fig. 2B). Starting from heterozygous founder animals, a colony of homozygous knock-in allele carriers and a colony of outbred wildtype control animals were established.

### Expression of Alox15 in peritoneal lavage cells is not altered by Leu353Phe in vivo mutagenesis

To explore whether the Leu353Phe in vivo exchange impacts the expression of different Alox-isoforms qRT-PCR was carried out for all seven mouse Alox-isoforms (Alox15, Alox15b, Alox12, Alox12b, Aloxe3, Aloxe12, Alox5) in peritoneal lavage cells. As expected from the results shown in Fig. 2A Alox15 mRNA is present at high quantities in these cells but we did not observe significant differences when the two genotypes were compared (Fig. 2C, left panel). In contrast, for Alox5 we found that expression of this enzyme was two fold lower in humanized *Alox15*-KI mice than in the outbred wildtype control peritoneal lavage cells (Fig. 2C, right panel). It should be stressed at this point that expression of Alox5 in peritoneal lavage cells was more than ten-fold lower than that of Alox15. Expression levels of other Alox-isoforms (Alox15b, Alox12, Alox12b, Aloxe3, Aloxe12) were below the detection limits of our assay system (Fig. 2C, middle panel). Taken together, these RT-PCR data indicate that Alox15 and Alox5 are the two major Alox-isoforms expressed in mouse peritoneal lavage cells and that wildtype and mutant Alox15 are expressed at similar levels. In other words, Leu535Phe exchange in the *Alox15* gene did not alter the expression efficiency of this enzyme but reduced the expression level of the pro-inflammatory Alox5 in peritoneal lavage cells.

### In vivo Leu353Phe exchange humanized the functional properties of mouse Alox15

**Fig. 3:**
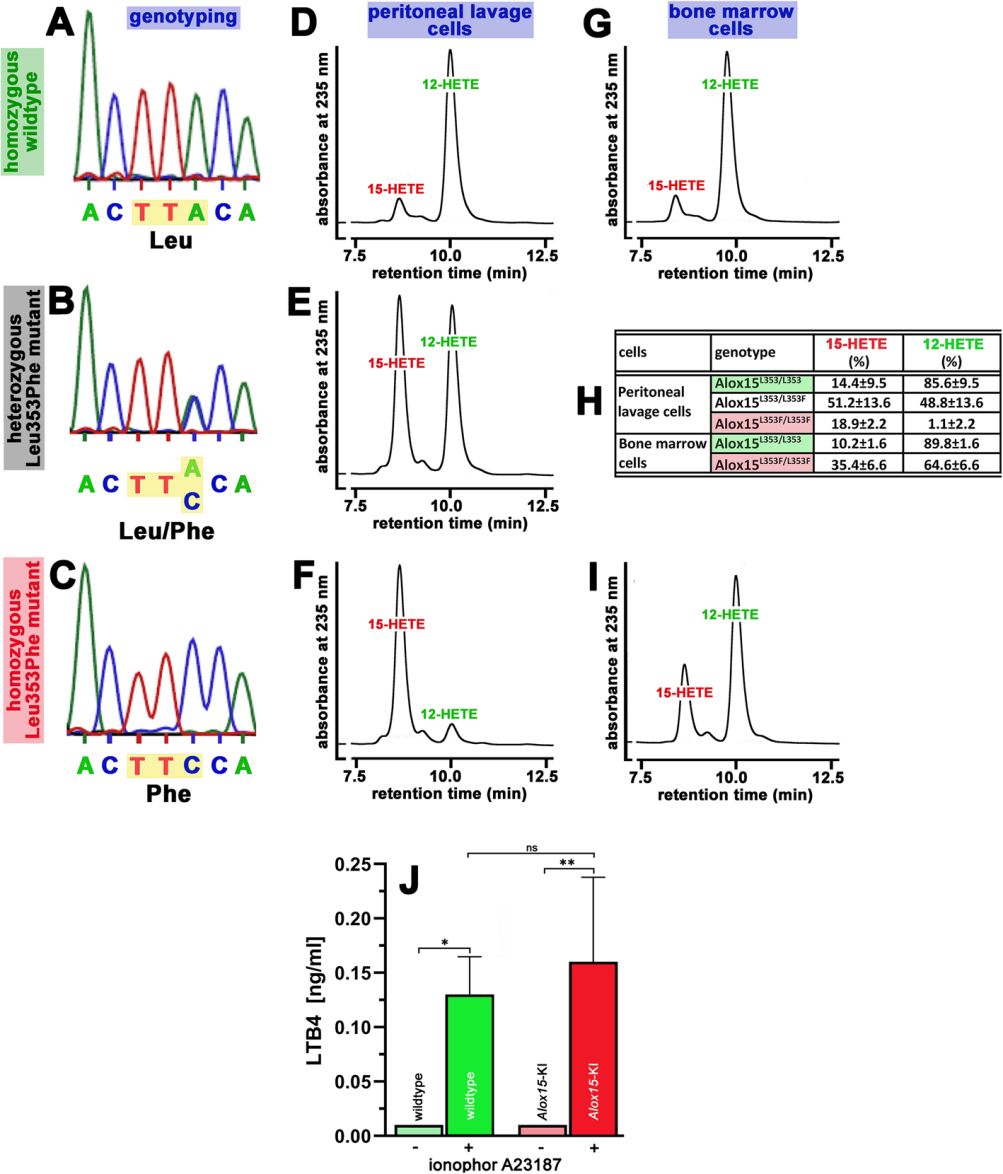
Genomic sequencing and ex vivo Alox activity assays. **A**–**C** The mutated region of the Alox15 gene was amplified by PCR and the amplification products were sequenced (for methodological details see Additional file 1). **D**–**F** Ex vivo Alox15 activity assays (see Additional file 1) using peritoneal lavage cells as enzyme source. Representative partial RP-HPLC chromatograms are shown. **G**, **I** Ex vivo Alox15 activity assays (see Additional file 1) using bone marrow cells as enzyme source. **H** Summary and statistical evaluation (means ± SD, *n* = 4) of the Alox15 activity assays. **J** Ex vivo Alox5 activity assays (*n* = 4) using whole blood as enzyme source. Leukotriene B4 formation was quantified as readout parameter

To test whether Leu353Phe in vivo exchange converted the reaction specificity of mouse Alox15 from major AA 12-lipoxygenation by the wildtype enzyme to dominant AA 15-lipoxygenation by the Leu353Phe mutant we again prepared peritoneal lavage cells from *Alox15*-KI mice and outbred wildtype control animals and carried out ex vivo activity assays. For these experiments, we selected three individuals from our *Alox15*-KI mouse colony, three representatives of outbred wildtype controls and three heterozygous allele carriers and genotyped these individuals (ear biopsies). Then we prepared peritoneal lavage cells and performed ex vivo activity assays. From Fig. 3A it can be seen that the PCR fragment obtained by genomic PCR of outbred wildtype control mice involved the TTA triplet, which encodes for Leu353. In contrast, the heterozygous mice involved both the TTA (wildtype, Leu) and TTC (mutant, Phe) triplets (Fig. 3B). For the homozygous mutant mice only the TTC triplet (Phe) was observed (Fig. 3C). These data defined the corresponding animals as homozygous mutant allele carriers.

Next, ex vivo activity assays were carried out with peritoneal lavage cells and with bone marrow cells prepared from the selected individuals. As indicated in Fig. 3D peritoneal lavage cells of wildtype mice converted AA predominantly to 12-HETE. Smaller amounts 15-HETE were also detected. These data are consistent with previous analytical data published for the AA oxygenation products of mouse peritoneal lavage cells [10, 11]. When peritoneal lavage cells of heterozygous allele carriers were employed a 1:1 mixture of 12-HETE and 15-HETE was formed (Fig. 3E). Such product pattern was expected when both, the wildtype (TTA, Leu) and the mutant (TTC, Phe) alleles are co-dominantly expressed in these cells. When homozygous *Alox15*-KI mice were used for the ex-vivo activity assays 15-HETE was analyzed as major oxygenation product (Fig. 3F). These activity data do not only show that Leu535Phe exchange humanized the reaction specificity of mouse Alox15 but they also confirm our qRT-PCR data (Fig. 2C) suggesting that there is hardly any difference in the expression levels of the wildtype and the mutant Alox15 alleles.

Taken together, these ex-vivo activity data indicated that our *in-vivo* mutagenesis strategy humanized the functional properties of mouse Alox15 when AA is used as oxygenation substrate. To confirm this conclusion for other cell types, we performed similar activity assays with bone marrow cells. As for wildtype peritoneal lavage cells we identified 12-HETE as dominant AA oxygenation product when bone marrow cells of outbred wildtype mice were used as enzyme source (Fig. 3G). Here again, small amounts of 15-HETE were also observed and the 12-HETE/15-HETE ratio was about 9:1 (Fig. 3H). For homozygous *Alox15*-KI mice, we quantified a 12-HETE/15-HETE ratio of about 2:1 (Fig. 3I). The most plausible explanation for this product pattern is that in addition to Alox15 other Alox isoforms such as Alox12 may contribute to 12-HETE formation in bone marrow cells. In fact, qRT-PCR studies indicated that in addition to Alox15 and Alox5 mRNA, Alox12 mRNA was found at high levels in bone marrow cells.

### Leu353Phe exchange in the *Alox15* gene did hardly impact the Alox5 pathway

In mouse blood Alox15 and Alox5 are co-expressed in leukocytes [8, 33] and thus, alterations of the Alox15 properties might impact the functionality of the Alox5 pathway. The two enzymes compete for the same substrate and the reactions products of the modified Alox15 pathway may alter the catalytic activity of Alox5. To test whether systemic humanization of the reaction specificity of Alox15 might alter the Alox5 pathway we quantified leukotriene B4 (LTB4) formation in whole blood following stimulation of the blood cells with calcium ionophore 23187. From Fig. 3J it can be seen that only small amounts of the Alox5 product LTB4 were formed from endogenous substrate when whole blood was incubated in the absence of calcium ionophore. In contrast, after A23187 stimulation large amounts of LTB4 were detected. A similar situation was observed when blood of *Alox15*-KI mice was used. Here again, small amounts of LTB4 were analyzed when blood was incubated in the absence of A23187. When A23187 was present, large amounts of the Alox5 product LTB4 were detected. However, there was no significant difference when LTB4 formation of the two genotypes was compared. Thus, humanization of the reaction specificity of Alox15 did not alter the Alox5 pathway of whole blood cells.

### Reproduction characteristics of Alox15-KI mice

Alox15 has previously been implicated in spermatogenesis [34] and thus, we tested the reproduction kinetics of *Alox15*-KI mice. Here we found that these animals reproduce normally. We compared litter size (pups per litter), frequency of pregnancy (litters per female x month), number of pups per female and month and the gender ratio of the newborns of Alox15-KI mice and wildtype control animals (Additional file 1: Fig. S1), but did not observe significant differences between the two genotypes. Thus, on the basis of this data it can be concluded that *Alox15*-KI mice are fully fertile and do not show major defects during embryogenesis.

### Body weight development

Comparing the bodyweight kinetics of male and female *Alox15*-KI mice with those of outbred wildtype controls we did not observe distinct growth behaviors of the two genotypes (Additional file 1: Fig. S2). The growth curves were largely superimposable for either sex over the entire experimental period and no significant differences were observed. Thus, functional humanization of mouse Alox15 does not significantly impact post-natal development of the genetically modified individuals.

### Blood plasma oxylipidome profiles revealed differences in the pattern of oxylipins between Alox15-KI mice and outbred wildtype controls

To test whether Leu353Phe exchange in mouse *Alox15* might have changed the plasma oxylipin patterns we profiled more than 40 different oxylipins by LC–MS/MS (see Additional file 1: Methodological supplement including Tables S3–S6). Some previously characterized oxylipins such as a number of maresin, resolvin and protectin isomers were below the detection limits of our analytical systems but for other oxylipins, we obtained reliable analytical data (Additional file 1: Figs. S3–S7).

**Fig. 4:**
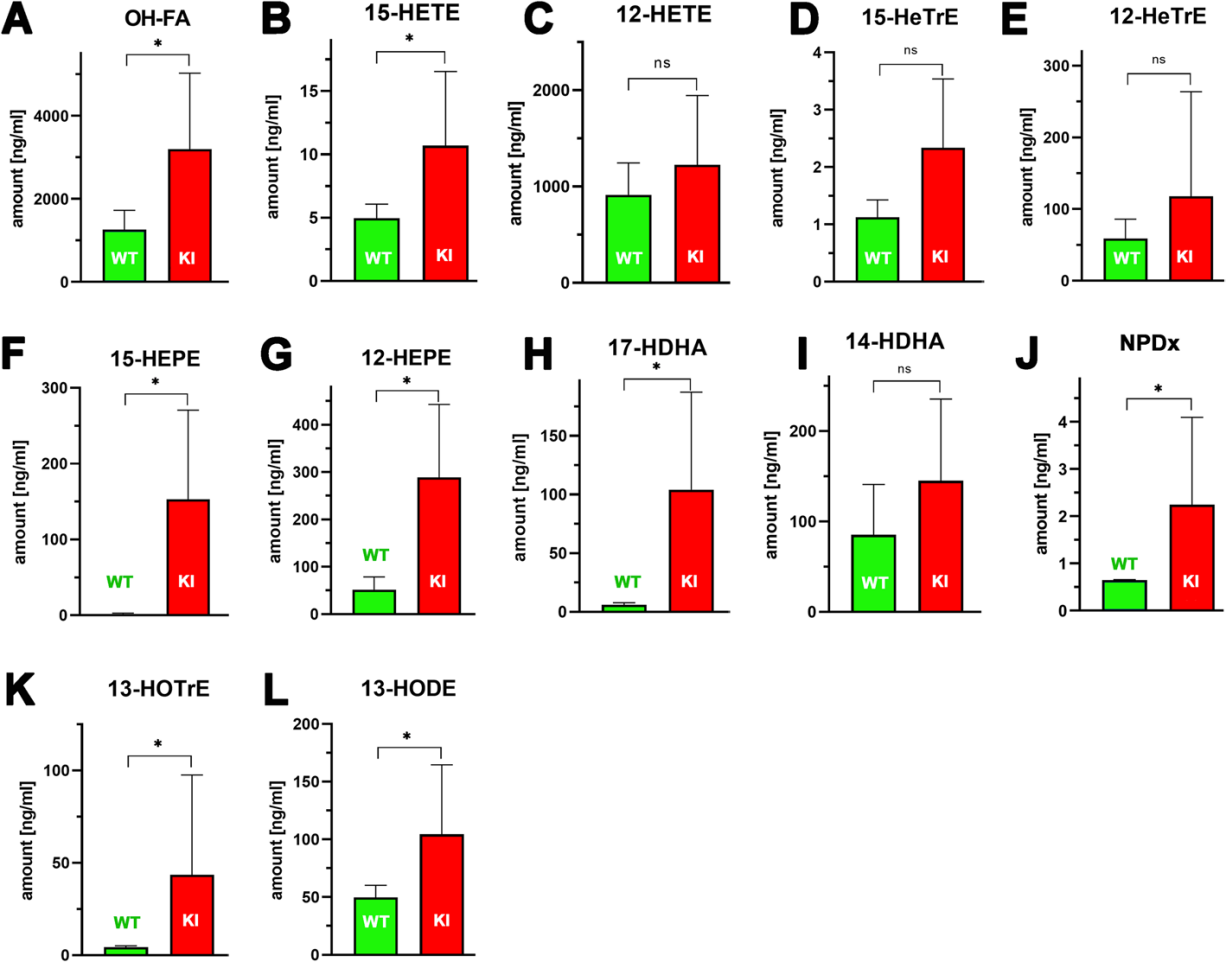
LC–MS based analysis of plasma oxylipidomes. Selected oxylipins were quantified (LC–MS/MS) in the blood plasma (see Additional file 1: for methodological details) of male *Alox15-*KI mice and of outbred wildtype controls (*n* = 5). Quantification of other oxylipins is given in Additional file 1: Figs. S3–S7. For statistical evaluation (n.s., not significant, *p < 0.05) the Mann–Whitney U-test was used

Comparing the whole amounts of oxylipins between the two genotypes, significantly more oxygenated PUFA derivatives were detected in *Alox15-*KI mice (Fig. 4A). Especially the levels of 12-HETE (Fig. 4C), 12-HETrE (Fig. 4E), 12- and 15-HEPE (Fig. 4F, G), 17- and 14-HDHA (Fig. 4H, I), as well as 13-HODE (Fig. 4L) were elevated in the plasma of *Alox15*-KI mice. Although for some of these metabolites non-significant differences were observed between the two genotypes for the sum of them the difference was statistically significant (Fig. 4A).

The dominant AA oxygenation product of mouse Alox15 is 12S-HETE, whereas the humanized enzyme produces mainly 15-HETE (Fig. 1A). As functional consequence of the Leu353Phe exchange we expect elevated plasma levels of 15-HETE but reduced plasma concentrations of 12-HETE in *Alox15*-KI mice. Although we observed significantly elevated 15-HETE concentrations in the plasma of *Alox15*-KI mice, the 12-HETE levels were not significantly different (Fig. 4B, C). These data suggest that the catalytic activity of Alox15 may not strongly contribute to the 12-HETE plasma levels in wildtype mice but that humanization of the reaction specificity of the enzyme is mirrored in the plasma by elevated 15-HETE concentrations.

It should be stressed at this point that the plasma 12-HETE concentrations are more than two orders of magnitude higher than the 15-HETE levels. If one assumes that half of the plasma 15-HETE originates from the Alox15 pathway, humanization of the reaction specificity of this enzyme would not significantly lower the blood plasma 12-HETE levels. Thus, the finding that we did not observe a significant reduction in the plasma 12-HETE concentrations is consistent with the obtained experimental data. However, it remains to be explored in the future which metabolic processes might contribute to the relatively high plasma 12-HETE levels (see Discussion).

For 12- and 15-HETrE we observed a similar situation (Fig. 4D, E). The plasma levels of 12-HETrE were almost two orders of magnitude higher than those of 15-HETrE and mouse Alox15 may not significantly contribute to the in vivo biosynthesis of 12-HETrE. Humanization of the reaction specificity of Alox15 induced elevated plasma concentrations of 15-HETrE and although the increase did not reach the level of statistical significance the humanized Alox15 might contribute to this increase.

12-HEPE and 12-HETrE were present at similar concentrations in the blood plasma of wildtype mice and humanization of the reaction specificity of Alox15 strongly (3–fourfold) elevated the 12-HEPE concentrations (Fig. 4E, G). This increase must be an indirect effect since humanization of the reaction specificity of the enzyme impaired its 12-HEPE synthase activity (Fig. 1B). The 15-HEPE plasma levels were strongly elevated (more than one order of magnitude) in *Alox15*-KI mice (Fig. 4F) and these data are consistent with the results obtained for the recombinant enzyme (Fig. 1B).

For the DHA metabolites (17-HDHA, 14-HDHA) we observed similar differences as for the EPA metabolites. Here again, the 14-HDHA plasma concentrations were higher in *Alox15*-KI mice (Fig. 4I) but the difference was statistically not significant. The plasma levels of 17-HDHA (Fig. 4H) were tenfold higher in *Alox15*-KI mice and this result may be related to the elevated 17-HDHA oxygenase activity of the humanized enzyme (Fig. 1D).

Neuroprotectins form a family of dihydroxylated derivatives of DHA, which exert potent anti-inflammatory, anti-apoptotic and neuroprotective activities [35, 36]. When we quantified the plasma oxylipins of *Alox15*-KI mice and outbred wildtype controls, we detected significant amounts of NPD-x in wildtype mice (Fig. 4J). Interestingly, much higher quantities of these metabolites were found in the plasma of *Alox15*-KI mice (Fig. 4J). If these compounds exhibit anti-inflammatory properties in vivo*, Alox15*-KI mice might be protected from inflammation in animal disease models. This conclusion can be tested in future research using the *Alox15*-KI mice described in this study.

The plasma levels of 13-HOTrE and 13-HODE (Fig. 4K, L) were significantly elevated in *Alox15*-KI mice but it remains unclear whether the humanized Alox15 might contribute to this effect.

In summary, our lipidomic data suggest that Leu353Phe exchange in mouse Alox15 induces alterations in the plasma oxylipin concentrations. A number of these differences might be a direct consequence of the functional alterations induced by this in vivo mutagenesis (elevated levels of 15-HETE, 15-HEPE and 17-HDHA in *Alox15*-KI mice) but other effects (elevated levels of 12-HEPE in *Alox15*-KI mice) are more indirect.

### Hematological parameters of Alox15-KI mice

**Fig. 5:**
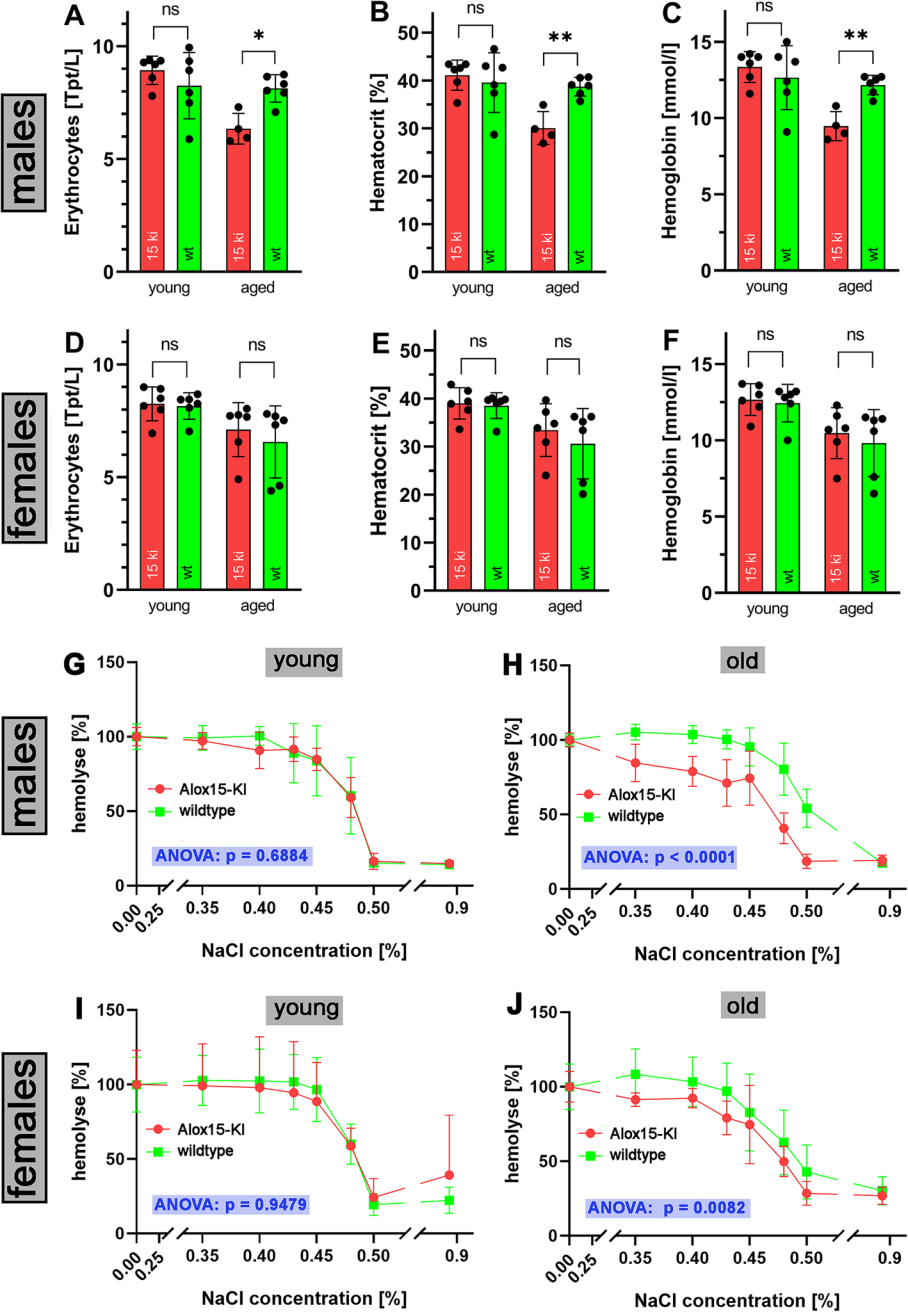
The erythropoietic system of Alox15-KI mice is compromised. **A**–**F** Erythrocyte parameters of *Alox15*-KI mice and outbred wildtype controls were determined in two age categories (young mice, 10–20 weeks; aged mice, 70–75 weeks, *n* = 6 for each age-group, Mann–Whitney U-test. ns, not significant, *p < 0.05, **p < 0.01.). Additional hematological parameters are given in Additional file 1: Fig. S6, S7. **G**–**J** Osmotic stability of erythrocytes: the osmotic stability of red blood cells in the two age categories (young mice, 10–20 weeks; aged mice, 70–75 weeks, *n* > 3 for each age-group) were determined as described in the Additional file 1. The degree of hemolysis was calculated at each NaCl concentration. Hemolysis curves were compared with two-way ANOVA

Alox15 has previously been implicated in erythropoiesis [19, 20] and detailed characterization of the hematological parameters of *Alox15*^*−/−*^ mice recently suggested that these animals carry a slightly dysfunctional erythropoietic system [22]. In fact, erythrocyte counts, hematocrit and hemoglobin concentrations of aged male *Alox15*^*−/−*^ mice were significantly lower than the corresponding values of outbred wildtype controls. Moreover, the osmotic stability and the ex vivo life span of *Alox15*^*−/−*^ erythrocytes were impaired [22]. Overexpression of human ALOX15 rescued this defective erythropoietic phenotype [22] and this data convincingly demonstrate that *Alox15*^*−/−*^ mice carry a slightly defective erythropoietic system. To characterize the erythropoietic systems of the two genotypes, selected blood parameters were determined in young (10–20 weeks) and aged mice (70–75 weeks) of either sex. Here we found that in aged male *Alox15*-KI mice the erythrocyte count (erys), the hematocrit (HK) and the hemoglobin (Hb were significantly (*p* < 0.01) were modified (Fig. 5A–C, right pairs of columns). For young males (Fig. 5A–C, left pair of columns) as well as for female individuals of either age category (Fig. 5D, E) such differences were not observed. For other hematological parameters we did not observe significant differences between *Alox15*-KI mice and corresponding wildtype controls (Additional file 1: Figs. S8, S9).

### Osmotic stability of red blood cells

Because of their high hemoglobin concentrations and because of the water permeability of their plasma membrane erythrocytes take up water when suspended in hypotonic solutions. Since this water uptake is quite excessive, the cells swell, hemolyze and release intracellular hemoglobin into the extracellular fluid. To compare the osmotic stability of peripheral erythrocytes prepared from *Alox15-*KI mice and outbred wildtype controls we prepared the peripheral red blood cells from corresponding individuals of either genotype and incubated them in phosphate buffer (pH 7.4) containing different concentrations of NaCl. The osmotic stability of the cells in the presence of different salt concentrations depends on the integrity of the plasma membrane and can be quantified by the degree of hemolysis [37]. From Fig. 5G, I it can be seen that erythrocytes prepared from young male and female individuals show almost superimposable hemolysis curves and thus, there was no difference in the osmotic stability of the red blood cells of the two genotypes. However, for aged individuals the hemolysis curves of the wildtype mice were shifted to the right (Fig. 5H, J) and these data indicate that at a given NaCl concentration the degree of hemolysis for wildtype erythrocytes was significantly (*p* < 0.0001 for males, *p* = 0.0082 for females) higher than that for *Alox15*-KI red blood cells. From these data, it can be concluded that erythrocytes of aged wildtype mice are more susceptible for osmotic challenge when compared with *Alox15*-KI red blood cells.

## Discussion

### In vivo Leu353Phe exchange humanized the reaction specificity of mouse Alox15

With AA as substrate mouse [10, 11] and human [38, 39] ALOX15 orthologs exhibit different reaction specificities and this catalytic difference might impact the in vivo functionality of the two orthologous enzymes. Detailed investigations of the reaction specificity of mammalian ALOX15 orthologs suggested that 95% of these enzymes are AA 12-lipoxygenating proteins [17, 18]. Only less than 5% of all mammalian species, which include all *Hominidae*, express AA 15-lipoxygenating isoenzymes. Gibbons are transition mammals since the ALOX15 orthologs of several gibbon subspecies exhibit a pronounced dual reaction specificity [17, 18]. Thus, the reaction specificity of ALOX15 orthologs has systematically been altered during late mammalian evolution (evolutionary concept of ALOX15 specificity [17, 18]). The driving force for the evolutionary change in reaction specificity remains unclear but it has previously been suggested that this alteration might be part of an evolutionary optimization of the immune system [17, 18]. If this concept is correct the humanized *Alox15-KI* mice should be protected in mouse inflammation models and we are currently testing this hypothesis for the DSS-induced colitis model, in the CFA-induced paw edema model and in different mouse atherosclerosis models. In the present paper we describe the creation and basic physiological characterization of these mice so that other scientists can employ them for other mouse models of human diseases.

It should be stressed at this point that the *Alox15*-KI mice described here exhibit other functional properties than the transgenic *ALOX15* mice (*ALOX15*-TG) we generated recently [40]. In the *ALOX15*-TG mice the transgenic arachidonic acid 15-lipoxygenating human ALOX15 is expressed in addition to the endogenous arachidonic acid 12-lipoxygenating mouse enzyme. For the *Alox15*-KI mice we modified the native *Alox15* gene in such a way that the endogenous Alox15 catalyzes AA 15-lipoxygenation. There is no additional expression of an AA 12-lipoxygenating enzyme and this constellation mirrors the human situation more closely. Moreover, expression of the transgenic human ALOX15 in the *ALOX15*-TG mice is regulated by the aP2 promoter and thus, the cellular expression pattern of the transgenic enzyme is different than that of native and mutant (Leu353Phe) mouse Alox15. In fact, the transgenic human ALOX15 is expressed at high levels in adipocytes [40] whereas expression of the native enzyme is more or less restricted to myeloic cells.

The Triad Concept explains the structural basis for the variable positional specificity of mammalian ALOX15 orthologs [3, 13] and thus, it may serve as suitable model to explain the functional differences. This concept was originally developed for rabbit ALOX15 [13] but extensive mutagenesis studies on other mammalian ALOX orthologs indicated that it is applicable to all mammalian ALOX15 orthologs regardless whether these enzymes are AA 12- or AA 15-lipoxygenating [17, 18]. According to the Triad Concept, the AA 12-lipoxygenating mouse Alox15 can easily be converted to an AA 15-lipoxygenating enzyme by simple Leu353Phe exchange and in vitro mutagenesis studies on the recombinant enzyme confirmed this hypothesis [16]. To explore whether this mutagenesis strategy will also work in vivo we produced knock-in mice, which carry a minor (two nucleotides) mutation in the *Alox15* gene employing a modified Crispr/Cas9 procedure and performed ex vivo activity assays using peritoneal lavage cells and bone marrow cells as enzyme source (Fig. 3). When peritoneal macrophages of wildtype mice were incubated with arachidonic acid, we observed major 12-HETE formation (Fig. 3D) and this data is consistent with previous reports [10, 11]. In contrast, when peritoneal lavage cells prepared from *Alox15*-KI mice were used for identical incubations 15-HETE was dominantly formed (Fig. 3F). This data indicates that the A-to-C exchange in exon 8 of the *Alox15* gene induced in vivo humanization of the reaction specificity of mouse Alox15.

For the ALOX15B orthologs of mice and humans a similar difference in the reaction specificity has previously been reported. In fact, human ALOX15B is a free AA 15-lipoxygenating enzyme [41] whereas its mouse ortholog oxygenates AA predominantly to 8S-HETE [42, 43]. When Y603 and H604 were mutated to the amino acids present at these positions in human ALOX15B (Y603N + H604V) functional humanization of mouse Alox15b was observed [44]. We recently generated and functionally characterized *Alox15b* knock-in mice (*Alox15b*-KI), which express the Y603N + H604V mutant of mouse Alox15b and found that this in vivo mutagenesis strategy humanized the reaction specificity of mouse Alox15b [23].

### Codominant expression of wildtype and mutant Alox15 alleles in heterozygous allele carriers

According to our ex vivo activity assays (Fig. 3E) heterozygous *Alox15*-KI mice express both, wildtype and mutant alleles at similar levels (codominance). Thus, in peritoneal lavage cells neither of the two alleles blocks the expression of the other allele. Codominant expression is a well-known phenomenon that has previously been described for the A and B alleles of the AB0 blood group system [45]. Another example of codominance is the beta-thalassemia minor involving a mutant of the gene encoding for the hemoglobin β-chain [46]. The heterozygote (β^o^β mutant) exhibits codominance because both alleles produce roughly equal amounts of their respective proteins. In most vertebrates the major histocompatibility complex (MHC) involves a set of closely linked polymorphic genes encoding proteins which are of relevance for the adaptive immune system [47]. The MHC complex is highly variable and the structural diversity of the encoded proteins has different reasons: (i) polygeny (multiple genes in the MHC cluster), (ii) polymorphism (variability between different individuals of a given species), (iii) codominant expression (both sets of inherited alleles are expressed at similar levels). According to our ex vivo activity data, the two alleles of the Alox15 gene locus are also co-dominantly expressed and this finding suggests that the two proteins may fulfill similar cellular functions. If the mutant allele would be dysfunctional, expression of this allele should have been downregulated whereas expression of the wildtype allele might have been augmented. However, we found that the Leu353Phe Alox15 allele is expressed at similar levels as the wildtype allele and this data is consistent with the ex vivo activity assays carried out with peritoneal lavage cells (Fig. 3E). Unfortunately, because of time constrains we did not study codominant expression of the two alleles when the mice were challenged in different mouse models of human diseases but such experiments can be carried out in follow-up studies.

### Alox15-KI mice do not exhibit obvious phenotypic alterations

The biological role of ALOX15 in humans and mice is still a matter of discussion and several recent reviews summarize the current hypotheses [5–7]. *Alox15*^*−/−*^ mice are viable, reproduce well and develop normally [11]. Similar results were obtained for the *Alox15*-KI mice described in this report (Additional file 1: Figs. S1, S2). However, when challenged in a number of animal disease models phenotypic differences have been reported when *Alox15*^*−/−*^ mice and wildtype control animals were compared [48–53]. One of the classical functions of Alox15 is its role in late red blood cell development [19, 54]. This putative role was later on challenged since *Alox15*^*−/−*^ mice did not develop severe anemia [11]. However, we recently reported that aged *Alox15*^*−/−*^ mice suffer from mild hyperchromic anemia with elevated reticulocyte counts [22]. This mildly defective erythropoietic phenotype could be rescued by transgenic expression of human ALOX15 [22]. When we explored the functionality of the erythropoietic system of the *Alox15*-KI mice, we did not observe any signs of malfunction in young animals. In contrast, aged males exhibited significantly reduced erythrocyte counts (Fig. 5A) as well as impaired hematocrits (Fig. 5B) and hemoglobin concentrations (Fig. 5C). Interestingly, such differences were not observed for female individuals (Fig. 5D–F). ALOX15 has previously been implicated in the maturational breakdown of mitochondria during reticulocyte differentiation [19, 54] and this function has been related to the capability of this enzyme to oxygenate the phospholipids of mitochondrial membranes [21, 55]. Such activity has been reported for both, AA 12- [56] and AA 15-lipoxygenating [21] ALOX15 orthologs but it remains to be explored, which of the two enzyme classes exhibits a higher membrane oxygenase activity.

### Plasma oxylipidomes mirror the subtle genetic manipulation of the Alox15 gene

To explore the in vivo activity of the genetically modified Alox15 we compared the blood plasma oxylipidomes of the *Alox15*-KI mice with those of outbred wildtype controls. If the AA 12-lipoxygenating wildtype Alox15 significantly contributes to the plasma 12-HETE levels, our genetic manipulation was expected to reduce the 12-HETE plasma concentrations whereas the 15-HETE levels should be augmented. We indeed found (Fig. 4B) that the 15-HETE plasma levels in our *Alox15*-KI mice were significantly elevated but we did not detect an anti-parallel reduction of 12-HETE. The most probable explanation for this observation is that in wildtype mice Alox15 does not significantly contribute to the high (1 µg 12-HETE/ml) plasma levels of 12-HETE. In fact, when we compared the 12-HETE blood plasma levels of wildtype C57BL/6 mice with those of Alox15 knockout animals (Alox15^−/−^) we did not find significant differences. Thus, other AA 12-lipoxygenating enzymes, most probably the Alox12 that is high level expressed in blood platelets, is responsible for the high blood plasma 12-HETE levels. Other AA 12-lipoxygenating Alox-isoforms, such as the epidermal Alox12b and/or Aloxe12 may not significantly contribute to the plasma 12-HETE levels since their expression in blood cells is very limited.

On the other hand, our plasma lipidome data (Fig. 4) also indicate that in addition to wildtype and mutant Alox15 other lipid oxidizing processes, such as fatty acid auto-oxidation or transition-metal catalyzed lipid peroxidation may contribute to the formation of plasma oxylipins. Moreover, kinetic differences between wildtype mouse Alox15 and its Leu353Phe mutant may also be considered as possible reasons for the differences in the plasma oxylipin concentrations of *Alox15-KI* mice and the outbred wildtype controls. In other words, lower 12-HETE plasma levels in the *Alox15*-KI mice are consistent with the interpretation that Alox15 may be involved in the biosynthesis of this metabolite but the data do not prove this interpretation. Similarly, higher 15-HETE levels in the plasma of Alox15-KI mice (compared with wildtype controls) do not prove that this metabolite originates from the Alox15 pathway but the findings are consistent with this interpretation. Unfortunately, for the time being little is known on the kinetic differences between wildtype mouse Alox15 and its Leu353Phe mutant and thus, no detailed discussion of this topic is possible.

### Erythrocytes of aged Alox15-KI mice exhibit a higher ex vivo osmotic resistance

Quantification of the osmotic stability of erythrocytes prepared from wildtype and *Alox15*-KI mice revealed an augmented osmotic resistance for the cells prepared from aged *Alox15*-KI mice (Fig. 5H, J). In fact, at each tested NaCl concentration the degree of hemolysis was lower for *Alox15*-KI erythrocytes when compared with wildtype cells. This observation seems to be not in agreement with our conclusion that the erythropoietic system of *Alox15*-KI mice might be compromised. In fact, one would expect that *Alox15*-KI erythrocytes are less resistant when exposed to osmotic stress. However, an increased osmotic resistance of the *Alox15*-KI erythrocytes might extend the life span of the cells and thus, may compensate the attenuated erythropoietic capacity of the bone marrow of *Alox15*-KI mice. We did not investigate if *Alox15*-KI erythrocytes have a longer in vivo life span since corresponding experiments exceed the frame of this study.

## Conclusion

Human and mouse ALOX15 orthologs form different product patterns when oxygenating polyenoic fatty acids. Here we describe that the reaction specificity of the mouse enzyme can completely be humanized in vivo by a single point mutation (Leu353Phe exchange) in the *Alox15* gene. For this study we generated knock-in mice that express the arachidonic acid 15-lipoxygenating Alox15 Leu353Phe mutant (*Alox15*-KI mice). These mice are fully fertile and develop normally. However, when compared with outbred wildtype control animals the *Alox15*-KI mice carry a slightly dysfunctional erythropoietic system. Since Alox15 may play important roles in the pathogenesis of inflammatory, hyperproliferative, metabolic and neurodegenerative diseases the *Alox15*-KI mice can now be studied in different mouse models of such human diseases.

### Supplementary Information

**Additional file 1.** Experimental and Methodological Supplement.

## Data Availability

All data generated during this study are included in this published article and/or in its supplementary information files. Original experimental raw data can be obtained upon request from the corresponding author.

## Abbreviations

AA: Arachidonic acid
ALOX: Arachidonic acid lipoxygenase
15-H(p)ETE: 15-Hydro(pero)xy-5*Z*,8*Z*,11*Z*,13*E*-eicosatetraenoic acid
12-H(p)ETE: 12-Hydro(pero)xy-5*Z*,8*Z*,10E,14*Z-*eicosatetraenoic acid
5-H(p)ETE: 5-Hydro(pero)xy-6*E*,8*Z*,11*Z*,14*Z*-eicosatetraenoic acid
15-H(p)EPE: 15-Hydro(pero)xy-5*Z*,8*Z*,11*Z*,13*E*,17*Z*-eicosapentaenoic acid
12-H(p)EPE: 12-Hydro(pero)xy-5*Z*,8*Z*,10E,14*Z*,17*Z-*eicosapentaenoic acid
14-H(p)DHA: 14-Hydro(pero)xy-4Z,7Z,10Z,12E,16Z,19Z-docosahexaenoic acid
17-H(p)DHA: 17-Hydroxy-4Z,7Z,10Z,13Z,15E,19Z-docosahexaenoic acid
13-H(p)ODE: 13-Hydro(pero)xy-9*Z*,11*E*-octadecadienoic acid
13-H(p)TrODE: 13-Hydro(pero)xy-9*Z*,11*E,15Z*-octadecatrienoic acid
LC–MS/MS: Liquid chromatography-mass spectrometry / mass spectrometry
IPTG: Isopropyl-β-thiogalactopyranoside
BHT: Butylhydroxy toluene
LTB4: Leukotriene B4
20-HETE: 20-Hydroxy-5*Z*,8*Z*,11*Z*,14*Z*-eicosatetraenoic acid
14,15-DHET: 14,15-Dihydroxy-5Z,8Z,11Z-dihydroxyeicosatrienoic acid
9,10-DiHOME: 9,10-Dihydroxy-12Z-octadecenoic acid
12,13-EpOME: 12(13)Epoxy-9Z-octadecenoic acid
8,9-EET: 8,9-Epoxy-5Z,11Z,14Z-eicosatrienoic acid
PGE2: Prostaglandin E2
