# Supplementary material for: Knock-in mice expressing a humanized arachidonic acid 15-lipoxygenase (Alox15) carry a partly dysfunctional erythropoietic system
Source: Cell Mol Biol Lett. 2023 Nov 29;28:97. doi: 10.1186/s11658-023-00511-3 (PMC10685687; doi:10.1186/s11658-023-00511-3)

**Additional file to the paper**

**Knock-in mice expressing a humanized arachidonic acid 15-lipoxygenase (Alox15) carry a partly dysfunctional erythropoietic system**

Florian Reisch^1,2,3^, Dagmar Heydeck^1^, Marjann Schäfer^1,2^, Michael Rothe^3^, Jiaxing Yang^1^, Sabine Stehling^1^, Gerhard P. Püschel^2^, and Hartmut Kuhn^1^*

^1^Charité – Universitätsmedizin Berlin, corporate member of Freie Universität Berlin and Humboldt Universität zu Berlin, Department of Biochemistry, Charitéplatz 1, D-10117 Berlin, Germany.

^2^Institute for Nutritional Sciences, University of Potsdam, Arthur-Scheunert-Allee 114-116, 14558 Nuthetal, Germany

^3^Lipidomix GmbH, Robert-Rössle-Straße 10, 13125 Berlin, Germany

**Running title**: *Alox15*-KI mice with humanized specificity

**Keywords:** eicosanoids, lipid peroxidation, oxidative stress, polyenoic fatty acids, erythropoiesis

**Corresponding author:** Dr. Hartmut Kuhn, Charité – Universitätsmedizin Berlin, corporate member of Freie Universität Berlin and Humboldt Universität zu Berlin, Department of Biochemistry, Charitéplatz 1, D-10117 Berlin, Germany. email: hartmut.kuehn@charite.de

**1. Methodological supplement**

*1.1. Bacterial expression of mouse Alox15 variants*

For expression of mouse Alox15 variants the coding region of the mouse *Alox15* gene was cloned into the bacterial expression plasmid pET28b. Competent bacteria [Rosetta2 DE3 pLysS] were transformed with 200 ng of the recombinant pET28b-Alox15 plasmid and cells were grown on kanamycin containing agar plates. Two well-separated bacterial clones were selected and 1 mL bacterial pre-cultures (LB medium with 50 μg/mL kanamycin and 35 μg/mL chloramphenicol) were grown at 37° C for 6 h at 180 rpm agitation. When the pre-culture reached an optical density (OD_600_) of 0.1-0.2 at a 1:50 dilution, the pre-culture was added to a 50 mL main culture. This main culture was grown overnight at 30° C in Ultra Yield flasks (Thomson Instrument Company, Oceanside, USA) covered with an air top seal (Fisher Scientific, Schwerte, Germany). The culture medium was freshly prepared from glucose-free minimal essential medium by the addition of trace elements and was supplemented with 40 g/L dextrin, 0.24 g/L tryptone/peptone and 0.48 g/L yeast extract. Immediately before starting the bacterial culture, antibiotics were added. Finally, 100 μL 1:20 diluted antifoam reagent 204 (Sigma, Deisenhofen, Germany) and 50 μL glycoamylase solution prepared from *Aspergillus niger* (Amylase AG 300L, Novozymes, Bagsværd, Denmark) were added to the culture medium. Expression of the recombinant enzymes was induced by the addition of 1 mM (final concentration) isopropyl-*β*-D-thiogalactopyranosid (IPTG) after the main culture reached an OD_600_ of 5. Additionally, 60 mg tryptone/peptone, 120 mg yeast extract, and 75–100 μL glucoamylase were added and the cultures were incubated for 24 h at 22° C and 230–250 rpm agitation. Bacteria were harvested by centrifugation and the resulting pellet was reconstituted in 5 mL PBS. Cells were lysed by sonication; cell debris was spun down and aliquots of the lysis supernatant were used for *in vitro* activity assays.

*1.2. Site directed mutagenesis of mouse Alox15*

For site-directed mutagenesis of recombinant mouse Alox15 10-50 ng plasmid-DNA were incubated with the specific primer pair (1 µL of 5 µM solution each) and 12.5 µL Pfu UltraI II Hot Start PCR Master Mix in a total volume of 25 µL adjusted with sterile water. To create the Leu353Phe mutant the following mutation primers were used: forward, 5‘-GTC CGA AGC TCA GAC TTC CAG CTT CAT GAG-3’; reversed, 3’- CTC ATG AAG CTG GAA GTC TGA GCT TCG GAC -5’ and PCR was performed as follows: 95°C for 2 min initial denaturation, cycle: 30 s at 95° C (denaturation phase), then 60 s at 55° C (annealing phase) followed by the synthesis phase (10 min at 68°C). This cycle was repeated 16 times. Subsequently the parent DNA was digested with 1 µL DpnI (Thermo Scientific, Schwerte, Germany) for 30 min at 37° C and the digestion was concluded by incubating the samples at 80° C for 10 min. 8 µL of the PCR sample were used for transformation of competent *E. coli* XL-1 Blue cells (Agilent Technologies Inc., Santa Clara, USA). After incubation for 30 min on ice, the cells were heat shocked for 45 s at 42° C, kept on ice for 2 min and then 400 µL SOC Medium was added. After 1 hr incubation at 37° C, the cells were plated on an LB-agar plate supplemented with 50 µg/mL kanamycin (for pET28b) and incubated overnight at 37° C. Four isolated growing clones were selected for liquid culture in 2 mL LB-Medium and plasmid DNA was prepared using the NucleoSpin Plasmid kit (Macherey & Nagel, Düren, Germany). One of these clones was selected for sequencing (Eurofins Genomics Germany GmbH, Ebersberg, Germany).

*1.3. In vitro activity assays of recombinant mouse Alox15 variants*

To determine the catalytic activity of the recombinant mouse Alox15 variants, variable amounts of the bacterial lysate supernatants were added to 0.5 mL of PBS containing arachidonic acid as substrate at a final concentration of 100 µM. After an 8 min of incubation period, the hydroperoxy compounds formed were reduced to the corresponding alcohols (addition of < 1 mg of solid sodium borohydride), the samples were acidified (35 µL of acetic acid), proteins were precipitated (addition of 0.5 mL of ice-cold acetonitrile) and precipitated proteins were removed by centrifugation. Aliquots of the protein-free supernatants (50-300 µL) were injected to reverse phase-HPLC analyses (RP-HPLC) to quantify the amounts of the conjugated dienes formed during the incubation period. A Shimadzu instrument (LC20 AD) equipped with a diode array detector (SPD M20A) was used and metabolites were separated on a Nucleodur C_18_ Gravity column (Macherey-Nagel, Düren, Germany; 250 x 4 mm, 5 μm particle size) coupled with a guard column (8 x 4 mm, 5 μm particle size). A solvent system consisting of acetonitrile : water : acetic acid (70:30:0.1, by vol) was employed at a flow rate of 1 ml/min and analytes were eluted isocratically at 25° C. When we analyzed the catalytic activity of the recombinant enzyme species, we always carried out no-enzyme control measurements, in which we used a lysate supernatant of *E. coli* cells which were transformed with an “empty” expression plasmid. In these control incubations we did not detect major amounts of ALOX products. In HPLC we observed small amounts of unspecific arachidonic acid auto-oxidation products but taken together they amounted to less than 5 % of the products detected in the enzyme incubation samples.

*1.4. Ex vivo Alox15 activity assays using peritoneal lavage and bone marrow cells*

To explore whether our *in vivo* mutagenesis strategy altered the reaction specificity of mouse Alox15, we carried out *ex vivo* activity assays using peritoneal lavage and bone marrow cells as enzyme sources. For this purpose, 0.5 ml of the cell suspensions were incubated for 5 min with 100 µM arachidonic acid. After incubation the primary AA oxygenation products were reduced with solid sodium borohydride, the samples were acidified (addition of 35 µL of concentrated acetic acid) and protein was precipitated by the addition of 0.5 ml ice-cold acetonitrile. Following 10 min of incubation on ice the protein precipitate was removed by centrifugation and aliquots of the protein-free supernatant were injected to RP-HPLC to analyze the oxygenated AA derivatives. As for the *in vitro* activity assays (see above) a Shimadzu instrument (LC20 AD) equipped with a diode array detector (SPD M20A) was used and the hydroxy fatty acids were separated on a Nucleodur C_18_ Gravity column (Macherey-Nagel, Düren, Germany; 250 x 4 mm, 5 μm particle size) coupled with a guard column (8 x 4 mm, 5 μm particle size). A solvent system consisting of acetonitrile : water : acetic acid (70:30:0.1, by vol) was employed at a flow rate of 1 ml/min and analytes were eluted isocratically at 25° C. The absorbance at 235 nm (absorbance maximum of the conjugated dienes) was recorded and uv-spectra of the dominant peaks were evaluated.

*1.5. Ex vivo Alox5 activity assay*

Heparinized blood was removed from sacrificed *Alox15*-KI mice and outbred wildtype controls (n=5 for each genotype). 200 µl of the whole blood were incubated for 15 min at 37° C in the absence or presence of 5 µM of calcium ionophore A23187, which activates the Alox5 pathway in polymorphonuclear granulocytes and other types of immune cells. This treatment activates intracellular phospholipases, which attack membrane phospholipids and liberate free AA that can subsequently be metabolized via the Alox5 pathway. After the incubation period the blood cells were removed by centrifugation and the plasma was quickly shock frozen in liquid nitrogen. After thawing the plasma lipids were extracted and the leukotriene B4 (LTB4) concentrations were quantified by LC-MS/MS.

*1.6. Generation of Alox15-KI mice*

The Alox15-KI mice characterized here were created in collaboration with Cyagen Bioscience (Santa Clara, USA). The mouse *Alox15* gene involves fourteen exons, with its ATG start codon located in exon 1. The target amino acid Leu353 was mapped to exon 8. The point mutation (Leu353Phe) was introduced into the wildtype mouse *Alox15* gene (GenBank accession number: NM_009660.3; Ensembl: ENSMUSG00000018924) that is located on mouse chromosome 11 using the Crispr/Cas9 mutagenesis strategy. The Leu353Phe exchange (TTA to TTC in exon 8) in the donor oligonucleotide was introduced by homology-driven repair. In addition to this nucleotide exchange, a silent mutation (CAG to CAA) was introduced to prevent the binding and re-cutting of the sequence by gRNA after homology-directed repair. Another silent mutation (CAG to CAA) was created in the construct for convenient MmeI restriction analysis during genotyping. Cas9 mRNA, the gRNA generated by *in vitro* transcription and the donor oligonucleotide were co-injected into fertilized eggs for the production of knock-in mice. Resulting pups were genotyped by genomic PCR followed by MemI restriction analysis and DNA sequencing. For *in vivo* mutagenesis the following gRNAs were designed: i) gRNA1 (matches the forward strand of the gene) CTT ACA GCT TCA TGA GCT ACA GG, ii) gRNA2 (matches reversed strand of the gene) GAA gct gta agt ctg agc ttc gg. PMA sequence is underscored. The following donor oligonucleotide sequence was used: ACG CCC TTG GAT CCC CCA ATG GAC TGG CTC CTG GCC AAA TGC TGG GTC CGA AGC TCA GAC **TTC** *CAA* CTT CAT GAG CTA *CAA* GCT CAT CTT CTG AGG GGA CAC TTG GTG GCT GAG GTC TTT GCT GTG GCC ACC A. The sequence of the target mutation (Leu353Phe) is underlined in bold and the silent mutations introduced are underlined in italic letters.

*1.7. Analysis of potential off-target mutations*

To exclude that off-target alterations have been introduced during our in vivo mutagenesis strategy we amplified genomic regions with similar sequences by genomic PCR and sequenced the PCR products. For all regions we obtained wildtype sequences **(Table S1)**.

**Table S1: Off-target analysis of our *in vivo* mutagenesis strategy for creating Alox15 knock-in mice.** When we screened the mouse reference genome with the sequence of our targeting oligonucleotide, we detected 5 potential off-target sequences that are localized on different chromosomes. These regions, which involved 4-5 nucleotide mismatches, were amplified by genomic PCR, the amplification products were prepared and subsequently sequenced. In all cases we detected the wildtype sequences and these data suggest that no off-target mutations were introduced during our in vivo mutagenesis strategy.

| **Location** | **Number of mismatches** | **Sequencing result** |
| --- | --- | --- |
| Chr11 18899612 - 118899634 | 4 | wildtype |
| Chr19 53769109 - 53769131 | 5 | wildtype |
| Chr11 12351891 - 12351913 | 5 | wildtype |
| ChrX 114820847 - 11482069 | 4 | wildtype |
| Chr1 90641020 - 90641042 | 5 | wildtype |

*1.8. qRT-PCR of Alox-isoforms in RNA extracts prepared from peritoneal lavage cells*

Total RNA was extracted from peritoneal lavage cells using the NucleoSpin RNA Plus kit (MACHEREY-NAGEL, Düren, Germany). Synthesis of cDNAs was performed with 0.2-0.8 µg of the total RNA preparations using oligo(dT) primers and Tetro Reverse Transcriptase (BIOLINE, Luckenwalde, Germany). Quantitative real-time PCR (qRT-PCR) was carried out with a Rotor Gene 3000 (Corbett Research Ltd., Saffron Walden, UK) started with a denaturation step (10 min at 95 ◦C). Afterwards, 40 cycles of amplification, each consisting of a denaturation (15 s at 95 ◦C), an annealing (30 s at 65 ◦C), and a synthesis period (20 s at 72 ◦C) were carried out using the Sensi-Fast SYBR PCR Kit (BIOLINE, Luckenwalde, Germany). Alox mRNA levels of the wildtype enzymes were set 100%. The gene specific amplification primers listed in **Table S2** were used (BioTez Berlin-Buch GmbH, Germany). The Rotor-Gene Q software was used to analyze the PCR raw data. Specific amplicons were prepared as amplification standards for all target (Alox isoforms) and reference (Gapdh) genes.

**Table S2. Sequences of the gene specific amplification primers used for qRT-PCR**

| Gene | Gene ID | Forward Primer 5’🡪3’ | Reverse Primer 5’🡪3’ |
| --- | --- | --- | --- |
| *Gapdh* | 14433 | CCATCACCATCTTCCAGGAGCGA- | GGATGACCTTGCCCACAGCCTTG |
| *Alox15* | 11687 | GTACGCGGGCTCCAACAACGA | TCTCCGGGGCCCTTCACAGAA |
| *Alox15b* | 11688 | CCTCCCGCTTATGTCTTTCCGT | GCCCTTTGACTTTCAGCTCCGTA |
| *Alox12* | 11684 | GCGGCCATGTTCAGTTGCTTAC | CATCGTCACGTCGTCCTTGCTG |
| *Alox12b* | 11686 | GGTGATGGTTCGGGGTCTGTCT | GAGTCCAGAGCACCAAGAGCACA |
| *Alox12e* | 11685 | CTCCAGCCACCACGACACGG | GCAACGAGTCCACAATGTCCCT |
| *Alox5* | 11689 | TCGAGTTCCCATGTTACCGCT | CTGTGGTCACTGGGAGCTTCG |
| *Aloxe3* | 23801 | GGGCGGCTATTGAGAGGTTTGT | TCTGGTCCTTTGGCTCTTGGCT |

*1.9. Blood plasma oxylipidomics*

For analysis of blood plasma oxilipins EDTA blood was drawn from sacrificed mice by heart puncture and the blood plasma was prepared by centrifugation. Then 10 µl of blood plasma were mixed with 450 µl of water and 10 µl of a mixture of internal standards (LTB4-d4, 20-HETE-d6, 15-HETE-d8, 13-HODE-d4, 14,15-DHET-d11, 9,10-DiHOME-d4, 12,13-EpOME-d4, 8,9-EET-d11, PGE2-d4; 10 ng/ml each). 5 µl of a butylhydroxytoluene (BHT) solution were added to prevent PUFA autooxidation during sample workup and storage. Plasma proteins were precipitated by the addition of 100 µl of a 1:4 mixture (by vol.) of glycerol/water and 500 µl acetonitrile. The pH was adjusted to 6.0 by the addition of 2 ml phosphate buffer (0.15 M), the precipitated proteins were removed by centrifugation and the clear supernatant was used for solid phase lipid extraction on a 200 mg Agilent Bond-Elute -Certify II cartridge (Agilent Technologies, Santa Clara, USA). Before sample application, the cartridge was conditioned with 3 ml methanol and 3 ml phosphate buffer (0.15 M, pH 6.0). After the sample was applied, the column was washed with 3 ml of a 1:1 mixture (by vol.) of methanol : water and the oxygenated fatty acids were eluted with a 74 : 25 : 1 mixture (by vol.) of ethyl acetate : n-hexane : acetic acid. The solvents were evaporated in a stream of nitrogen, the remaining lipids were reconstituted in 100 µl of a 6:4 mixture (by vol.) of methanol : water and used for LC-MS/MS analysis.

LC-MS/MS was carried out on an Agilent 1290/II LC-MS system consisting of a binary pump system, an autosampler and a column oven (Agilent Technologies, Waldbronn, Germany). We employed an Agilent Zorbax Eclipse C_18_ UPLC column (150 x 2.1 mm, 1.8 µm particle size) at 30° C. The solvent gradient was mixed of two stock solutions. Stock A: Water containing 0.05 % acetic acid. Stock B: 1:1 mixture (by vol.) of methanol : acetonitrile. The solvent gradient employed for our analyses is specified in **Table S3**. The quantified metabolites and their detection limits are listed in **Table S4**. To avoid chromatographic artefacts the injection system was rinsed after each injection with a 5 : 4 :1 mixture (by vol.) of methanol : water : isopropanol. The HPLC system was connected with a triple quadrupole MS system (Agilent 6495 System, Agilent Technologies, Santa Clara, USA). Negative electrospray ionization was carried out and the ionization parameters are given in **Table S5**. Each metabolite was detected simultaneously by two independent mass transitions **(Table S6)**. Metabolite-specific calibration curves were set up for each quantified metabolite. Experimental raw data were evaluated with the Agilent Mass-Hunter software, version B10.0.

**Table S3. Mobile phase gradient used for LC-separation of the plasma oxylipins.** The extracted plasma oxylipins were separated by RP-HPLC as described in the Material and Methods section using the following gradient of the mobile phase. The following solvent stock solutions were used: Stock A: water containing 0.05 % acetic acid (low elution power), stock B: 1:1 mixture of methanol : acetonitrile (high elution power).

| Time [min] | Solvent stock solution B [%] |
| --- | --- |
| 0 | 5 |
| 0.5 | 56 |
| 5.5 | 61 |
| 18.5 | 87 |
| 18.6 | 98 |

**Table S4: Metabolites quantified in the frame of our plasma oxylipidome analysis.**

| Parent PUFA | Metabolite | Detection limit (ng/ml plasma) |
| --- | --- | --- |
| 9,12-octadecadienoic acid | 9-HODE | 0.59 |
|  | 13-HODE | 0.30 |
| 9,12,15-octadecadienoic acid | 9-HOTrE | 0.86 |
|  | 13-HOTrE | 0.86 |
| 8,11,14-eicosatrienoic acid | 5-HeTrE | 0.21 |
|  | 8-HeTrE | 0.92 |
|  | 12-HeTrE | 0.49 |
|  | 15-HeTrE | 0.51 |
| 5,8,11,14-eicosatetraenoic acid | 5-HETE | 0.41 |
|  | 8-HETE | 3.12 |
|  | 12-HETE | 0.67 |
|  | 15-HETE | 0.51 |
|  | LTB4 | 0.28 |
|  | 5(S),12(S)-DiHETE | 0.41 |
|  | 5(S),15(S)-DiHETE | 0.25 |
|  | 8(S),15(S)-DiHETE | 0.35 |
| 5,8,11,14,17-eicosapentaenoic acid | 5-HEPE | 0.41 |
|  | 8-HEPE | 0.20 |
|  | 12-HEPE | 0.46 |
|  | 15-HEPE | 0.72 |
|  | 18-HEPE | 0.52 |
|  | RvE1 | 1.24 |
| 4,7,10,13,16,19-docosahexaenoic acid | 4-HDHA | 0.33 |
|  | 7-HDHA | 0.55 |
|  | 8-HDHA | 1.07 |
|  | 10-HDHA | 0.29 |
|  | 11-HDHA | 0.35 |
|  | 13-HDHA | 1.56 |
|  | 14-HDHA | 0.28 |
|  | 16-HDHA | 0.29 |
|  | 17-HDHA | 0.94 |
|  | 20-HDHA | 0.44 |
|  | 22-HDHA | 0.18 |
|  | Maresin 1 | 0.32 |
|  | Maresin 2 | 0.22 |
|  | Maresin 7-epi | 0.31 |
|  | RvD1 | 1.84 |
|  | RvD1 17(R) | 2.50 |
|  | RvD2 | 2.40 |
|  | RvD3 | 1.89 |
|  | RvD4 17(R,S) | 2.50 |
|  | RvD5 | 1.40 |
|  | NPD1 | 0.17 |
|  | NPD-x | 0.36 |

**Table S5: Ionization conditions used in our LC-MS based analysis of the blood plasma lipidomes.** The extracted plasma lipids were analyzed by LC-MS as described in the Materials and Method section and the ionization conditions used for MS analysis are specified in this table.

| Parameter | Conditions |
| --- | --- |
| Drying gas temperature / flow | 105 °C / 16 L/min |
| Sheath gas temperature / flow | 390 °C / 12 L/min |
| Nebulizer pressure | 32 psi |
| Capillary voltage | - 4300 V |
| Nozzel voltage | - 1950 V |

**Table S6. Characteristic mass transitions, collision energies and retention times of the different oxylipins.**

| Oxylipin | Precursor Ion | Product  Ion | MS Res | Collision Energy (V) | Ret Time (min) |
| --- | --- | --- | --- | --- | --- |
| 10-HDHA | 343.2 | 181.1 | Wide | 11 | 16.725 |
| 10-HDHA | 343.2 | 153.1 | Wide | 14 | 16.725 |
| 11-HDHA | 343.2 | 149.1 | Wide | 12 | 17.013 |
| 11-HDHA | 343.2 | 121.1 | Wide | 13 | 17.013 |
| 11-HETE | 319.2 | 167.1 | Wide | 14 | 16.623 |
| 11-HETE | 319.2 | 149.1 | Wide | 22 | 16.623 |
| 12-HEPE | 317.2 | 179.1 | Wide | 13 | 15.01 |
| 12-HEPE | 317.2 | 135.1 | Wide | 13 | 15.01 |
| 12-HETE | 319.2 | 179.2 | Wide | 13 | 16.935 |
| 12-HETE | 319.2 | 135.1 | Wide | 14 | 16.935 |
| 12-HeTrE | 321.2 | 181 | Wide | 20 | 17.9 |
| 12-HeTrE | 321.2 | 209 | Wide | 22 | 17.9 |
| 13-gamma-HOTrE | 293.2 | 193 | Wide | 18 | 14.46 |
| 13-HDHA | 343.2 | 221.2 | Wide | 10 | 16.546 |
| 13-HDHA | 343.2 | 193.2 | Wide | 12 | 16.546 |
| 13-HODE | 295.2 | 195.2 | Wide | 18 | 15.813 |
| 13-HODE-d4 | 299.2 | 198.1 | Wide | 18 | 15.857 |
| 13-HOTrE | 293.2 | 195 | Wide | 18 | 14.07 |
| 13-HOTrE | 293.2 | 223 | Wide | 20 | 14.07 |
| 14,15-DHET-d11 | 348.3 | 207.1 | Wide | 20 | 13.031 |
| 14,15-DHET-d11 | 348.3 | 140.1 | Wide | 20 | 13.031 |
| 14-HDHA | 343.2 | 205.1 | Wide | 11 | 16.734 |
| 14-HDHA | 343.2 | 161.1 | Wide | 13 | 16.734 |
| 15(R)epi-LXA4 | 351 | 135 | Wide | 18 | 7.186 |
| 15(R)epi-LXA4 | 351 | 115 | Wide | 15 | 7.186 |
| 15-HEPE | 317.2 | 219.2 | Wide | 11 | 14.648 |
| 15-HEPE | 317.2 | 175.1 | Wide | 13 | 14.648 |
| 15-HETE | 319.2 | 219.2 | Wide | 11 | 16.252 |
| 15-HETE | 319.2 | 121.1 | Wide | 16 | 16.252 |
| 15-HETE-d8 | 327.2 | 226.2 | Wide | 11 | 16.143 |
| 15-HETE-d8 | 327.2 | 214.2 | Wide | 14 | 16.143 |
| 15-HETE-d8 | 327.2 | 182.1 | Wide | 14 | 16.143 |
| 17(R)RvD1 | 375 | 215 | Wide | 18 | 7.227 |
| 17(R)RvD1 | 375 | 141 | Wide | 16 | 7.227 |
| 17(R,S)-RvD4 | 375.3 | 131 | Wide | 14 | 8.51 |
| 17(R,S)-RvD4 | 375.3 | 101 | Wide | 21 | 8.51 |
| 17-HDHA | 343.2 | 245.2 | Wide | 10 | 16.431 |
| 17-HDHA | 343.2 | 201.1 | Wide | 13 | 16.431 |
| 18-HEPE | 317.2 | 259.2 | Wide | 9 | 14.22 |
| 18-HEPE | 317.2 | 215.2 | Wide | 13 | 14.22 |
| 20-HDHA | 343.2 | 241.2 | Wide | 11 | 16.059 |
| 20-HDHA | 343.2 | 227.2 | Wide | 15 | 16.059 |
| 20-HETE-d6 | 325.2 | 295.2 | Wide | 18 | 15.045 |
| 20-HETE-d6 | 325.2 | 281.1 | Wide | 16 | 15.045 |
| 4-HDHA | 343.2 | 133.1 | Wide | 14 | 17.983 |
| 4-HDHA | 343.2 | 101 | Wide | 13 | 17.983 |
| 5(S),15(S)-DiHETE | 335.3 | 173 | Wide | 14 | 10.88 |
| 5(S),15(S)-DiHETE | 335.3 | 255.1 | Wide | 18 | 10.88 |
| 5(S),12(S)-DiHETE | 335.2 | 195.1 | Wide | 16 | 11.82 |
| 5(S),12(S)-DiHETE | 335.2 | 129 | Wide | 20 | 11.82 |
| 5-HEPE | 317.2 | 201.1 | Wide | 13 | 15.48 |
| 5-HEPE | 317.2 | 115.1 | Wide | 11 | 15.48 |
| 5-HETE | 319.2 | 191.2 | Wide | 14 | 17.521 |
| 5-HETE | 319.2 | 115.1 | Wide | 14 | 17.521 |
| 5-HeTrE | 321.2 | 115 | Wide | 14 | 19.64 |
| 5-HeTrE | 321.2 | 259 | Wide | 15 | 19.64 |
| 6(S)-LXA4 | 351 | 135 | Wide | 19 | 7.6 |
| 6(S)-LXA4 | 351 | 115 | Wide | 13 | 7.6 |
| 7-epi-MaR1 | 359 | 297 | Wide | 13 | 10.081 |
| 7-epi-MaR1 | 359 | 177 | Wide | 16 | 10.081 |
| 7-HDHA | 343.2 | 141.1 | Wide | 11 | 17.144 |
| 7-HDHA | 343.2 | 113.1 | Wide | 18 | 17.144 |
| 8,9-EET-d11 | 330.3 | 268.2 | Wide | 12 | 18.64 |
| 8,9-EET-d11 | 330.3 | 167.1 | Wide | 13 | 18.64 |
| 8,9-EET-d11 | 330.3 | 159.1 | Wide | 24 | 18.64 |
| 8-HDHA | 343.2 | 189.1 | Wide | 11 | 17.338 |
| 8-HDHA | 343.2 | 109 | Wide | 12 | 17.338 |
| 8-HEPE | 317.2 | 161.1 | Wide | 17 | 14.91 |
| 8-HEPE | 317.2 | 155.1 | Wide | 13 | 14.91 |
| 8-HETE | 319.2 | 155.1 | Wide | 12 | 16.915 |
| 8-HETE | 319.2 | 127.1 | Wide | 22 | 16.915 |
| 9-HEPE | 317.2 | 167.1 | Wide | 13 | 15.201 |
| 9-HEPE | 317.2 | 149.1 | Wide | 13 | 15.201 |
| 9-HETE | 319.2 | 151.1 | Wide | 13 | 17.299 |
| 9-HETE | 319.2 | 123.1 | Wide | 15 | 17.299 |
| 9-HODE | 295.1 | 171 | Wide | 21 | 15.94 |
| 9-HODE | 295.1 | 277 | Wide | 21 | 15.94 |
| 9-HOTrE | 293.2 | 171 | Wide | 13 | 13.88 |
| 9-HOTrE | 293.2 | 121 | Wide | 18 | 13.88 |
| LTB4 | 335.2 | 129 | Wide | 20 | 11.45 |
| LTB4 | 335.2 | 195.1 | Wide | 16 | 11.45 |
| LTB4-6-trans | 335.2 | 195.1 | Wide | 16 | 10.54 |
| LTB4-6-trans | 335.2 | 129 | Wide | 20 | 10.54 |
| LTB4-6-trans-epi | 335.2 | 195.1 | Wide | 16 | 10.89 |
| LTB4-6-trans-epi | 335.2 | 129 | Wide | 20 | 10.89 |
| LTB4-d4 | 339.2 | 197.1 | Wide | 16 | 11.59 |
| LTB4-d4 | 339.2 | 59 | Wide | 32 | 11.59 |
| LXA4 | 351 | 135 | Wide | 19 | 7.15 |
| LXA4 | 351 | 115 | Wide | 13 | 7.15 |
| LxA5 | 349 | 233 | Wide | 11 | 5.656 |
| LxA5 | 349 | 115 | Wide | 18 | 5.656 |
| LXB4 | 351 | 233 | Wide | 13 | 6.2 |
| LXB4 | 351 | 221 | Wide | 15 | 6.2 |
| Maresin 1 | 359 | 177 | Wide | 16 | 11.38 |
| Maresin 1 | 359 | 113 | Wide | 15 | 11.38 |
| Maresin 2 | 359.3 | 232.2 | Wide | 15 | 12.52 |
| Maresin 2 | 359.3 | 221.2 | Wide | 12 | 12.52 |
| NPD1 | 359.3 | 206.1 | Wide | 15 | 10.89 |
| NPD1 | 359.3 | 153 | Wide | 15 | 10.89 |
| NPDx | 359.2 | 206 | Wide | 16 | 10.76 |
| NPDx | 359.2 | 153 | Wide | 16 | 10.76 |
| PGE2-d4 | 355.2 | 275.2 | Wide | 18 | 6.185 |
| PGE2-d4 | 355.2 | 193.2 | Wide | 20 | 6.185 |
| RvD1 | 375 | 215 | Wide | 18 | 7.073 |
| RvD1 | 375 | 141 | Wide | 16 | 7.073 |
| RvD2 | 375 | 277 | Wide | 13 | 6.299 |
| RvD2 | 375 | 175 | Wide | 23 | 6.299 |
| RvD3 | 375 | 147 | Wide | 22 | 6.23 |
| RvD3 | 375 | 115 | Wide | 18 | 6.23 |
| RvD5 | 359 | 199 | Wide | 14 | 10.98 |
| RvD5 | 359 | 141 | Wide | 13 | 10.98 |
| RvE1 | 349 | 195 | Wide | 17 | 4.16 |
| RvE1 | 349 | 107 | Wide | 22 | 4.16 |

*1.10. Osmotic resistance of erythrocytes*

Dysfunctional erythrocytes rapidly undergo osmotic hemolysis **[20]** and we followed the experimental protocol described in **[18]** to quantify this readout parameter. In brief, 2 μl of EDTA-blood was diluted in 200 μl phosphate buffer (pH 7.4) containing NaCl at different concentrations (0-0.85%) and the cells were incubated for 30 min at room temperature. The samples were centrifuged at 200 x g for 10 min and the supernatant containing the free hemoglobin, which was released during hemolysis, was quantified assaying the absorbance at 540 nm. The absorbance values were expressed as a percentage of complete hemolysis, which was achieved when the cells were incubated in the absence of NaCl. This absorbance was set 100%.

**2. Supplemental experimental data**

**Figure S1: Comparison of fertility parameters of *Alox15*-KI mice and outbred wildtype controls.** For *Alox15*-KI mice 8 breeding pairs and for outbred wildtype controls 7 breeding pairs (one male + 2 females) were mated and the different fertility parameters (x-axis) were quantified over a breeding period of 11 (*Alox15*-KI mice) and 10 (wildtype controls) months, respectively. Statistic evaluation of the experimental raw data was carried out using the Mann-Whitney U-test but no significant differences were observed between the two genotypes. n.s., not significant. Means ± SD are plotted.

*~~
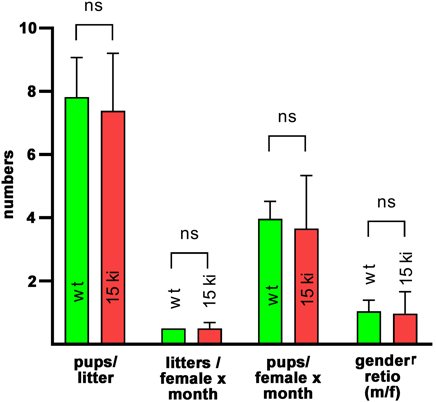
~~*

**Figure S2: Comparison of the bodyweight kinetics of *Alox15*-KI mice and outbred wildtype controls.** For each genotype, 10 individuals (n = 6) of either sex were housed in one cage with water and food (standard chow diet) *ad libitum*. Body weights were quantified once a week. A) Male individuals, B) Female individuals. Statistical evaluation of the experimental raw data was carried out with the two-way ANOVA.


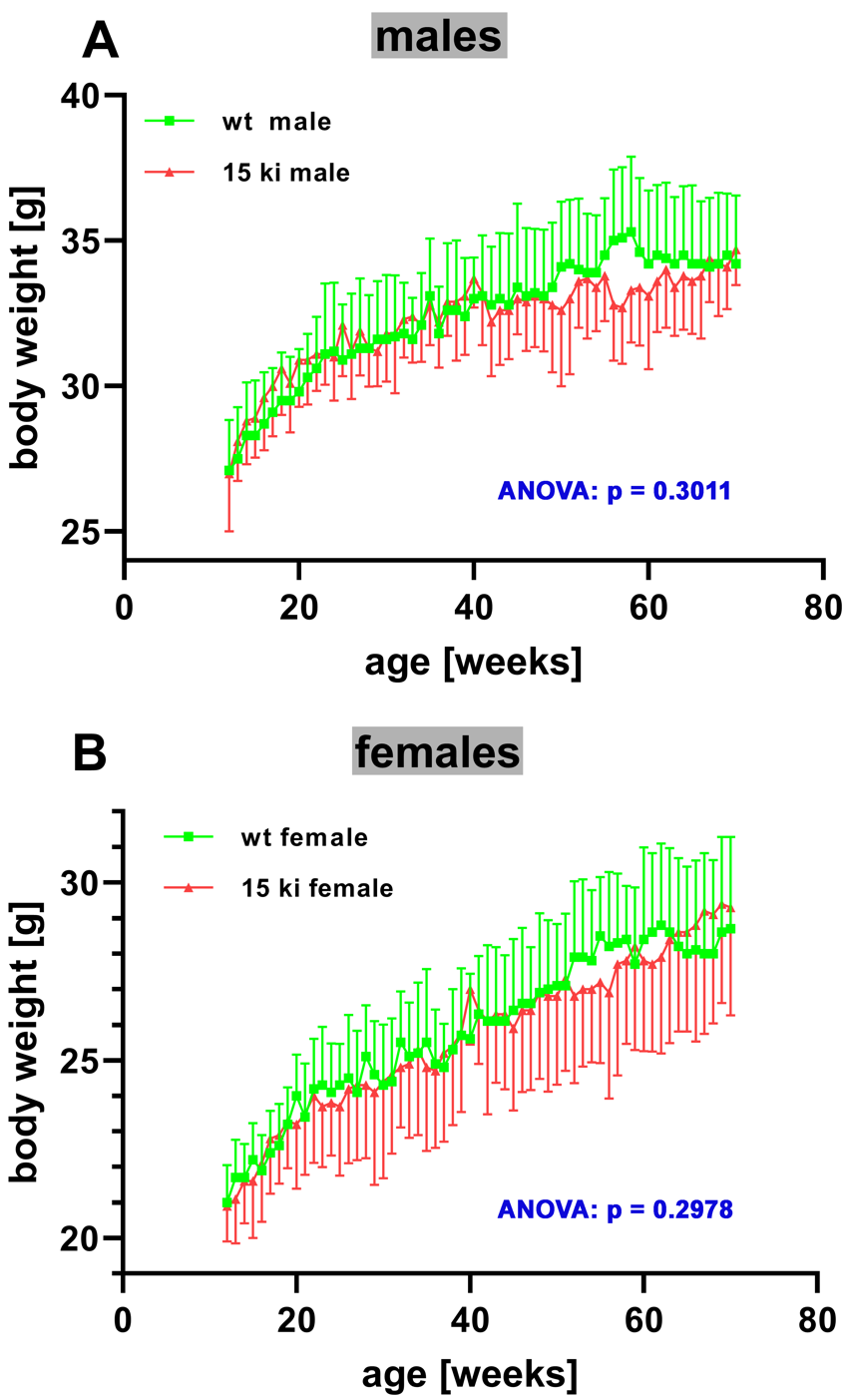


**Figure S3. Quantification of the major free oxygenated arachidonic acid metabolites in the blood plasma of *Alox15*-KI mice and outbred wildtype controls.** The lipids were extracted from the blood plasma of *Alox15*-KI mice and outbred wildtype controls and the specified free oxylipins were analyzed by LC-MS (see Materials and Methods).


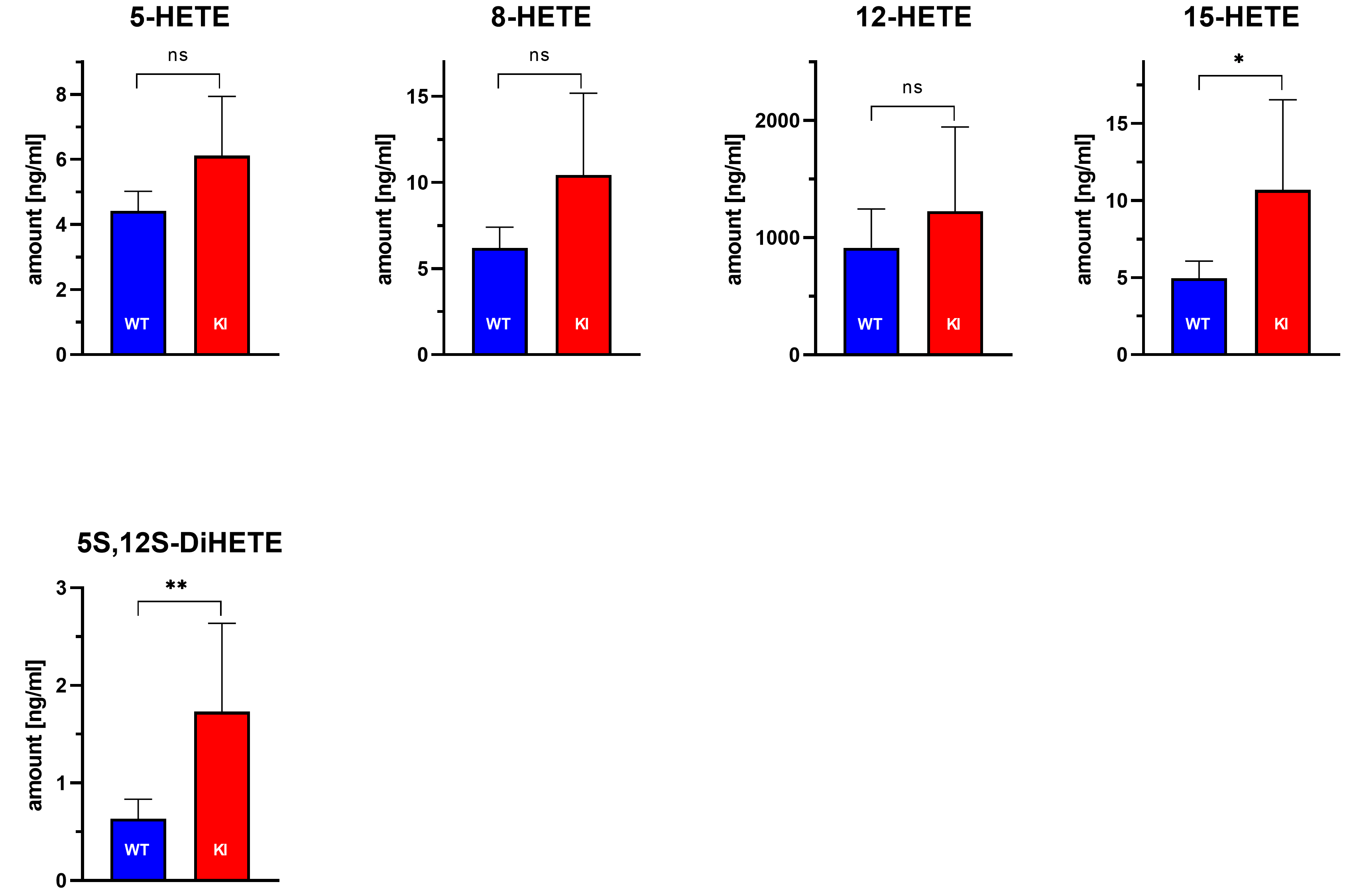


**Figure S4. Quantification of the major free oxygenated metabolites of 8,11,14-eicosatrienoic acid in the blood plasma of *Alox15*-KI mice and outbred wildtype controls.** The lipids were extracted from the blood plasma of *Alox15*-KI mice and outbred wildtype controls and the specified free oxylipins were analyzed by LC-MS (see Materials and Methods).


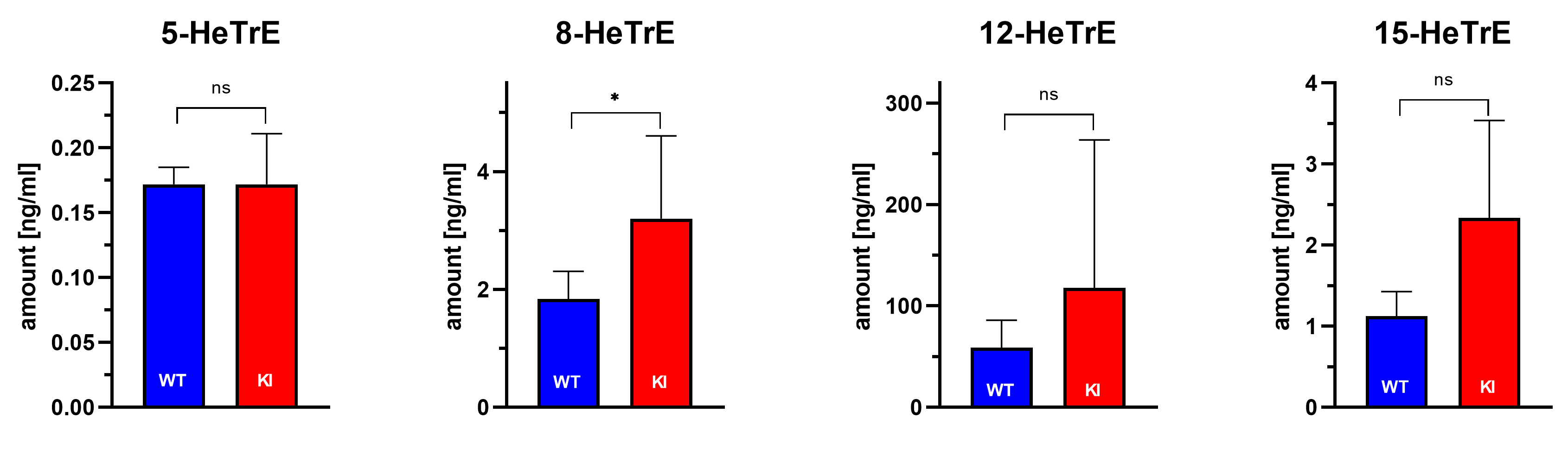


**Figure S5. Quantification of the major free oxygenated 5,8,11,14,17-eicosapentaenoic acid metabolites in the blood plasma of *Alox15*-KI mice and outbred wildtype controls.** The lipids were extracted from the blood plasma of Alox15-KI mice and outbred wildtype controls and the specified free oxylipins were analyzed by LC-MS (see Materials and Methods).


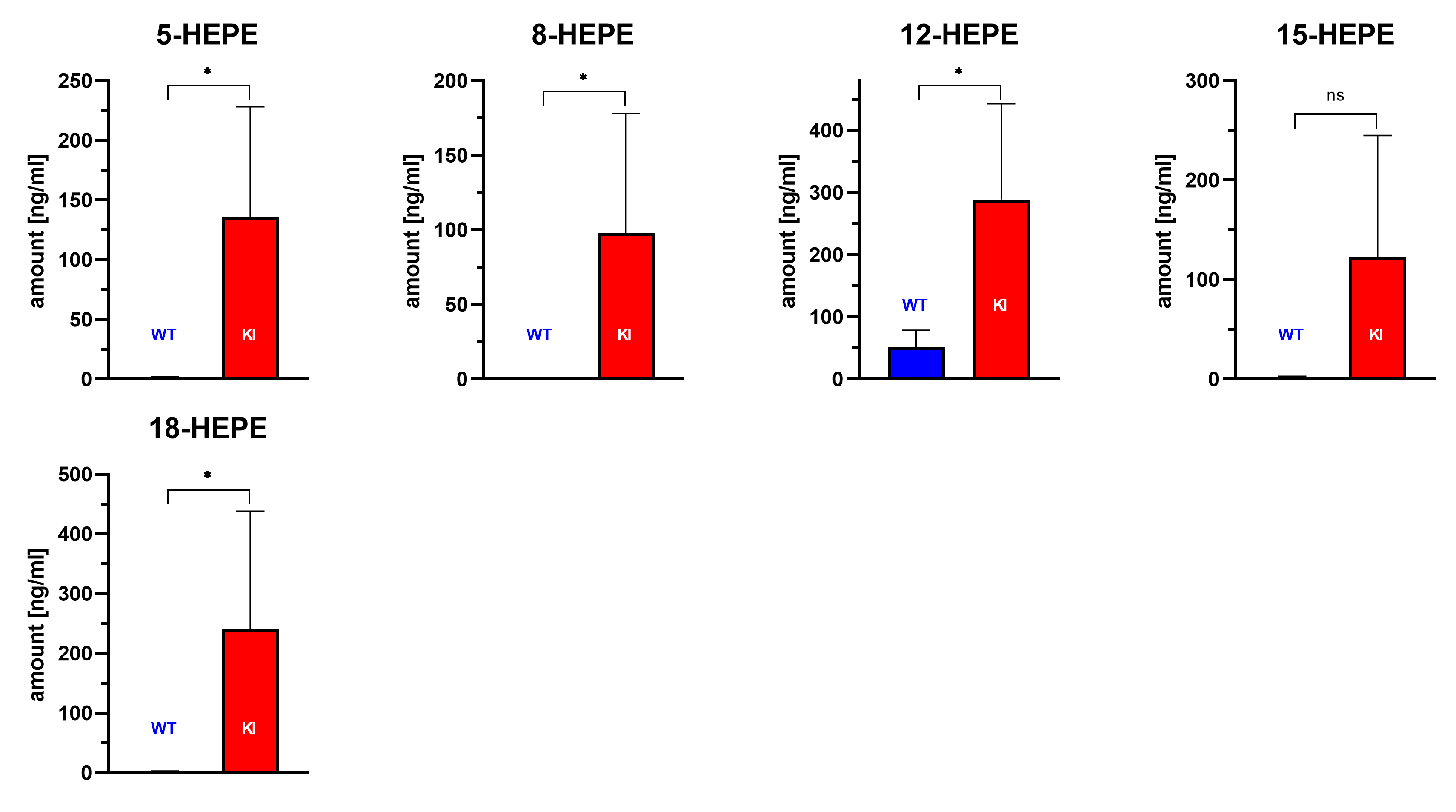


**Figure S6. Quantification of the major free oxygenated 4,7,10,13,16,19-docosahexaenoic acid metabolites in the blood plasma of *Alox15*-KI mice and outbred wildtype controls.** The lipids were extracted from the blood plasma of *Alox15*-KI mice and outbred wildtype controls and the specified free oxylipins were analyzed by LC-MS (see Materials and Methods).


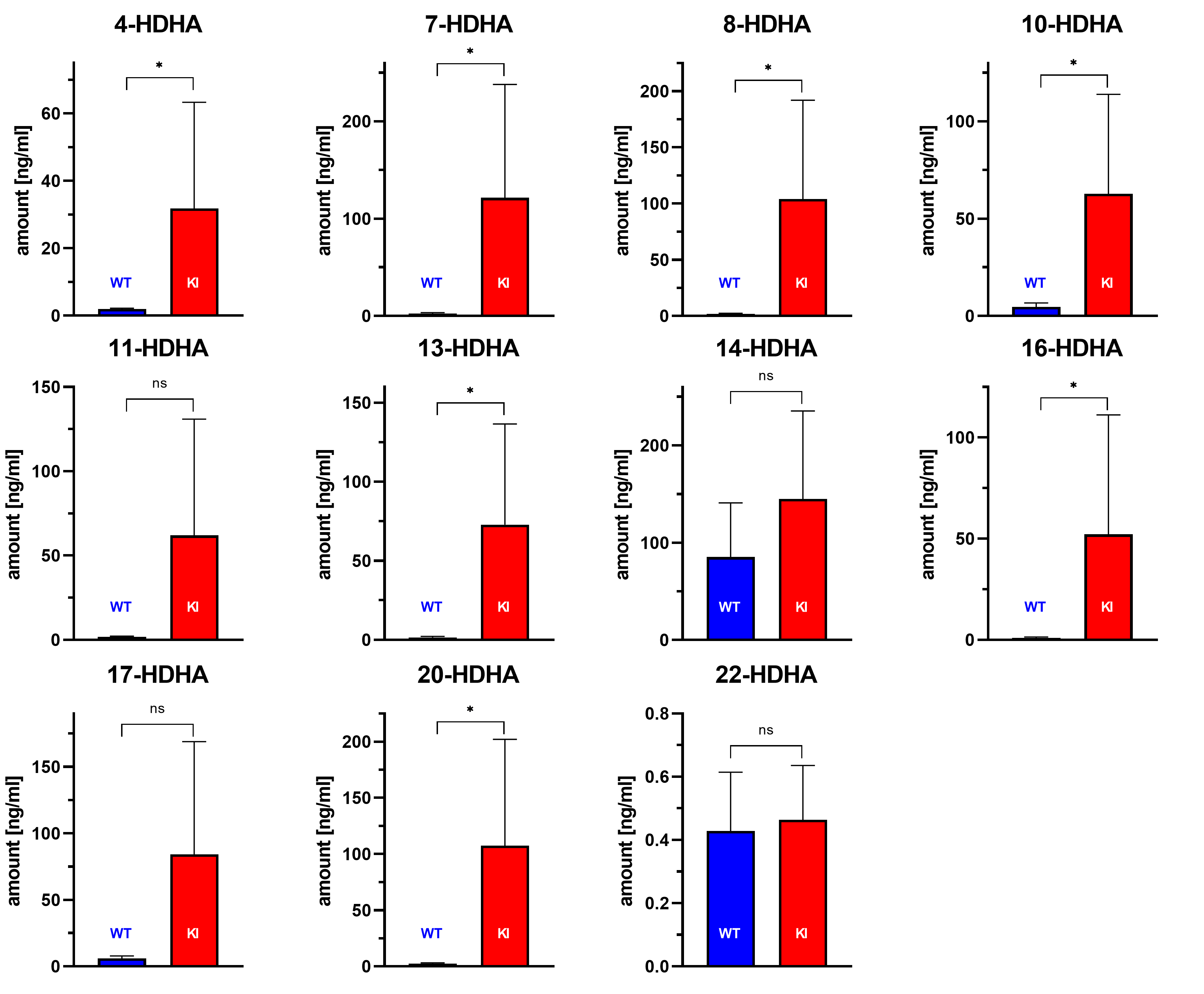


**Figure S7. Quantification of the major free oxygenated linoleic acid (HODE-isomers) and alpha-linolenic acid metabolites (HOTrE-isomers) in the blood plasma of *Alox15*-KI mice and outbred wildtype controls.** The lipids were extracted from the blood plasma of *Alox15*-KI mice and outbred wildtype controls and the specified free oxylipins were analyzed by LC-MS (see Materials and Methods).


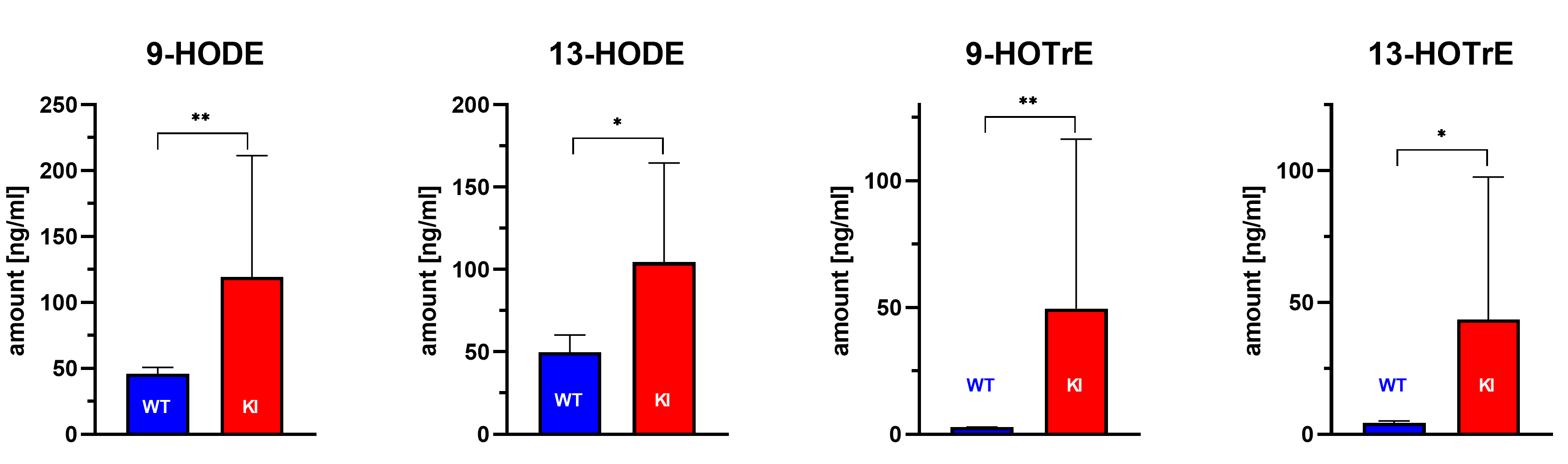


**Figure S8. Basic hematological parameters of male *Alox15*-KI mice and outbred wildtype control animals of different age categories.** Male *Alox15*-KI mice and outbred wildtype controls were classified in three age categories (young mice, 10-20 weeks; middle-aged mice, 30-40 weeks; old mice, 70-75 weeks, n=5 for each age-group). After sacrificing the animals by cervical dislocation under anesthesia EDTA blood was removed by heart puncture. The basic hematological parameters were determined by Institut für Veterinärmedizinische Diagnostik GmbH (Berlin, Germany).


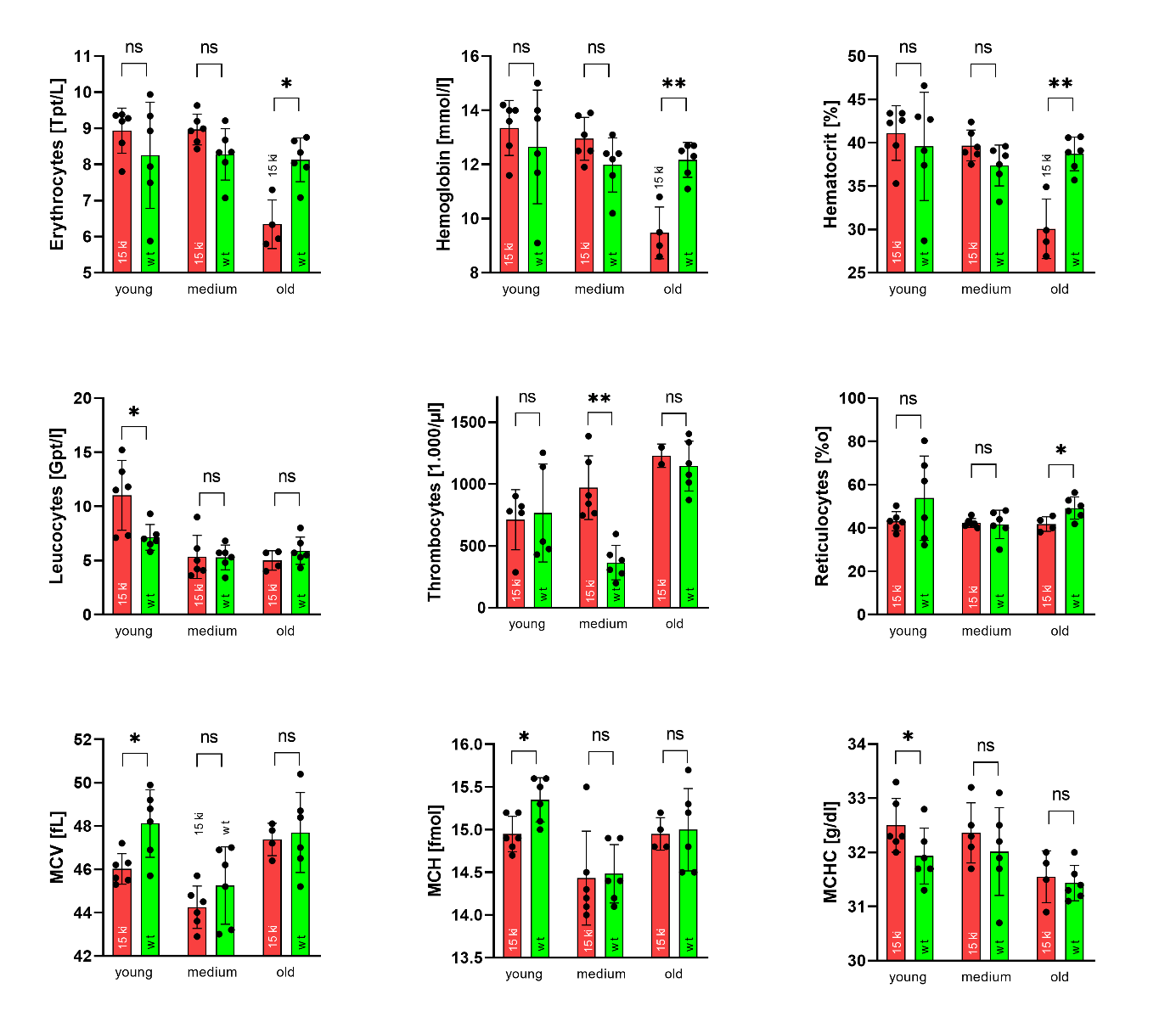


**Figure S9. Basic hematological parameters of female *Alox15*-KI mice and outbred wildtype control animals of different age categories.** Female *Alox15*-KI mice and outbred wildtype controls were classified in three age categories (young mice, 10-20 weeks; middle-aged mice, 30-40 weeks; old mice, 70-75 weeks, n=5 for each age-group). After sacrificing the animals by cervical dislocation under anesthesia EDTA blood was removed by heart puncture. The basic hematological parameters were determined by Institut für Veterinärmedizinische Diagnostik GmbH (Berlin, Germany).


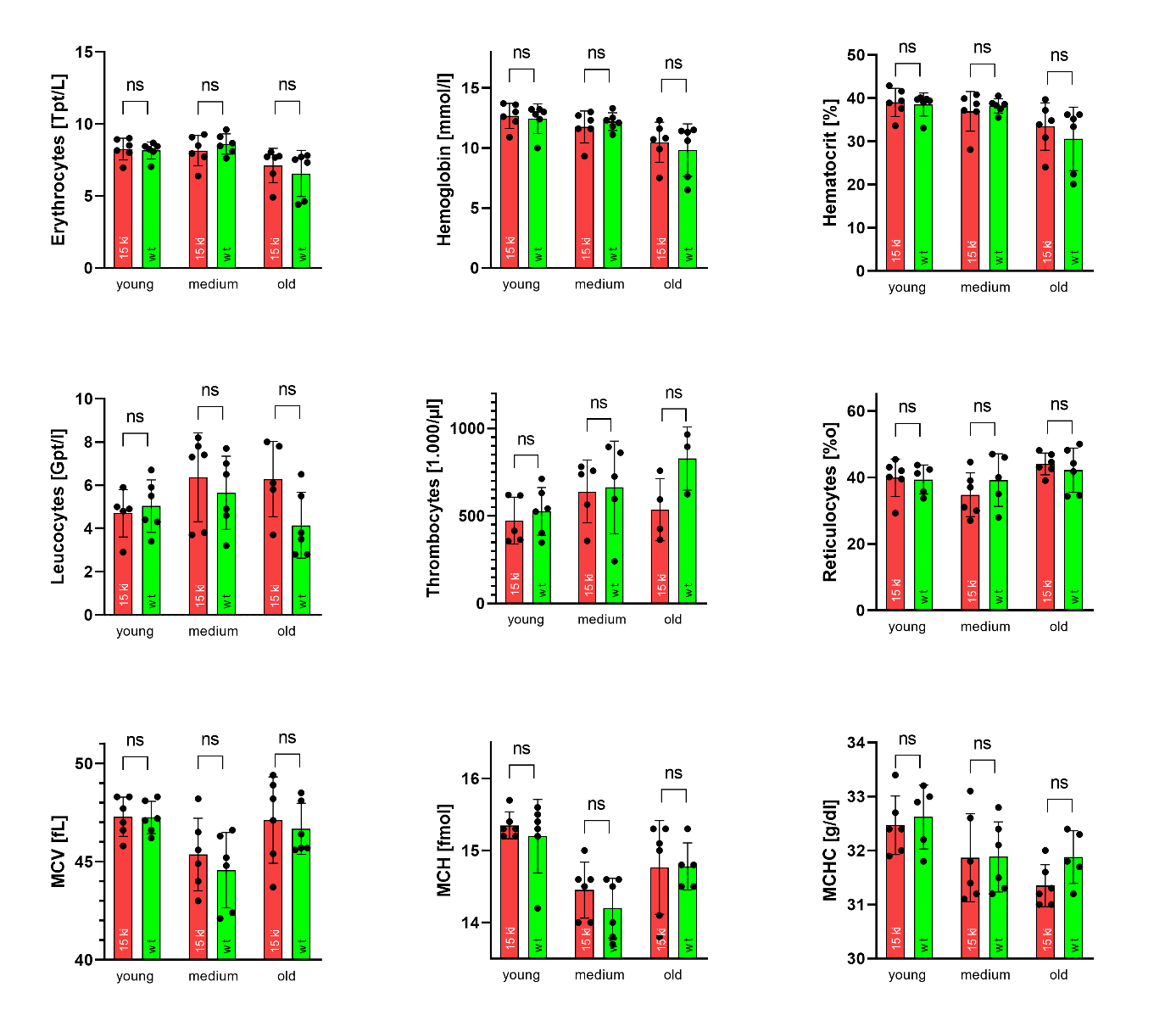

Supplement: Supplementary file 1 — Additional file 1. Experimental and Methodological Supplement. [file 11658_2023_511_MOESM1_ESM.docx]
